# Seed Germination Protocols (Standard, Accelerated Aging) and Plant Growth Traits for Wild *Schoenocaulon texanum* Scheele, Texas Feathershank (Melanthiaceae)

**DOI:** 10.3390/plants15152323

**Published:** 2026-07-28

**Authors:** Neil O. Anderson, Chase Mowry, Michele Schermann, Rajmund Eperjesi, Robert A. Suranyi, Darrick Unger, Steve Gullickson

**Affiliations:** 1 Department of Horticultural Science, University of Minnesota, 1970 Folwell Avenue, Saint Paul, MN 55108, USA; scher019@umn.edu (M.S.); rama.eperjesi@gmail.com (R.E.); 2 MCIA, Minnesota Crop Improvement Association, 1900 Hendon Ave, Saint Paul, MN 55108, USA; chase.mowry@mncia.org; 3 McLaughlin, Gormley King Co., LLC, and Sumitomo Botanicals, Minneapolis, MN 55428, USA; robert.suranyi@mgk.com (R.A.S.); darrick.unger@mgk.com (D.U.); steve.gullickson@mgk.com (S.G.)

**Keywords:** alkaloids, cevadine, green pesticides, saturated salt accelerated aging, soil testing, tunicate bulbs, veratridine

## Abstract

Green pesticides provide sustainable, biorational alternatives to synthetic pesticides. Sabadilla, *Schoenocaulon*, store alkaloids in their seeds; veratridine and cevadine are extracted with insecticidal activity. Wild, extant populations of native, N. American, *S. texanum*, are the focus of this research. The goal of this research was to determine soil constituents; alkaloid levels; quantify reproductive capacity for bulbs/plant, inflorescences/plant, seed capsules/plant, seeds/plant; seed dimensions, 100 seed wt.; standard and saturated salt accelerated aging (SSAA) percent seed germination of open-pollinated, untreated seed. We tested 10 genotypes and derived open-pollinated seed lots from five non-sympatric populations collected in Texas. Soil samples showed variation for all soil characteristics. Organic matter levels and micro-nutrients were low; pH = 7.8–8.2; heavy metals (Al, Cd, Cr, Ni, Pb) were detected. Seeds of *S. texanum* are smaller than *S. officinale*, but share appearance, hardness, color and shape. Alkaloid content was ~20% lower than *S. officinale*. Mean number of bulbs/clump was 2–12 with 1–8 flower stems/plant. Mean 100 seed weight = 0.45 g. No seeds germinated in weeks 1–2 (G1, G2) in standard or SSAA tests. Highest % germination in both tests was in G3–G4, declining precipitously in G5–G6. We establish initial seed germination standards of ≥70% for both standard and SSAA germination tests, based on total or adjusted final % germination of seed lots.

## 1. Introduction

The Melanthiaceae, bunchflower or corn lily family (formerly included within the Liliaceae), is a diverse higher plant family of monocotyledonous, geophytic, perennial angiosperms, native to the Americas and Eurasia in both hemispheres [1,2,3,4,5,6,7]. Most taxa contain various alkaloid compounds in above- and/or below-ground plant structures which are associated with naturally occurring sodium-channel toxins with insecticidal properties, veratridine and cevadine [8,9]. Genera within the family include: *Amianthium* A. Gray, *Anticlea* Kunth, *Chamaelirium* Willd., *Chionographis* Maxim., *Helonias* L., *Heloniopsis* A. Gray, *Melanthium* J. Clayton ex L., *Paris* L., *Pseudotrillium* S.B. Farmer, *Schoenocaulon* A. Gray, *Stenanthium* (A. Gray) Kunth, *Toxicoscordion* Rydb., *Trillium* L., *Veratrum, Xerophyllum* Michx., *Ypsilandra* Franch., and *Zigadenus* Michx. [1,2,3,5]. Most taxa contain various alkaloid compounds in above- and/or below-ground plant structures, such as bulbs and contractile roots (*Anticlea*, *Melanthium*, *Stenanthium*), rhizomes and roots (*Chamaelirium*, *Chionographis*, *Pseudotrillium*, *Trillium*, *Veratrum*, *Xerophyllum*), leaves (*Stenanthium*), seeds (*Melanthium*, *Schoenocaulon*) or all above- and below-ground parts (*Amianthium*, *Helonias*, *Heloniopsis*, *Paris*, *Toxicoscordion*, *Ypsilandra*, *Zigadenus*).

Species in the Melanthiaceae are of interest for development as potential crops [4,9]. Factors involved in domestication include a wide range of plant-specific traits as well as environmental factors of the native environment in which a designated taxon grows. Herein we review information for a *Schoenocaulon* species for such purposes. The center of origin for the genus *Schoenocaulon* is Mexico [1]. Wild populations of *Schoenocaulon* naturally occur in North America (Mexico, southern United States), Central America (Costa Rica, El Salvador, Guatemala, Honduras), and South America (Peru, Venezuela) [10,11]. All *Schoenocaulon* species are diploid (2*n* = 2*x* = 16) and colonizing perennial geophytes with distinctive fibrous-tunicate bulbs with dark brown to black scales and fibers, endemic to undisturbed sites [12]. *Schoenocaulon officinale* A. Gray ex Benth. (Schltdl. & Cham.), sabadilla, is an ancient green pesticide crop currently under domestication for commercial production [4,8,9,13,14,15,16]. *Schoenocaulon* is unique within the Melanthiaceae, storing the highest concentrations of steroidal alkaloids, naturally occurring sodium-channel toxins, veratridine and cevadine, in seeds [4,17,18], rather than underground storage organs, i.e., tunicate bulbs. Most *S. officinale* seeds are wild harvested sporadically from native stands on Venezuelan hillsides for extraction of veratridine and cevadine [8,9].

**Figure 1:**
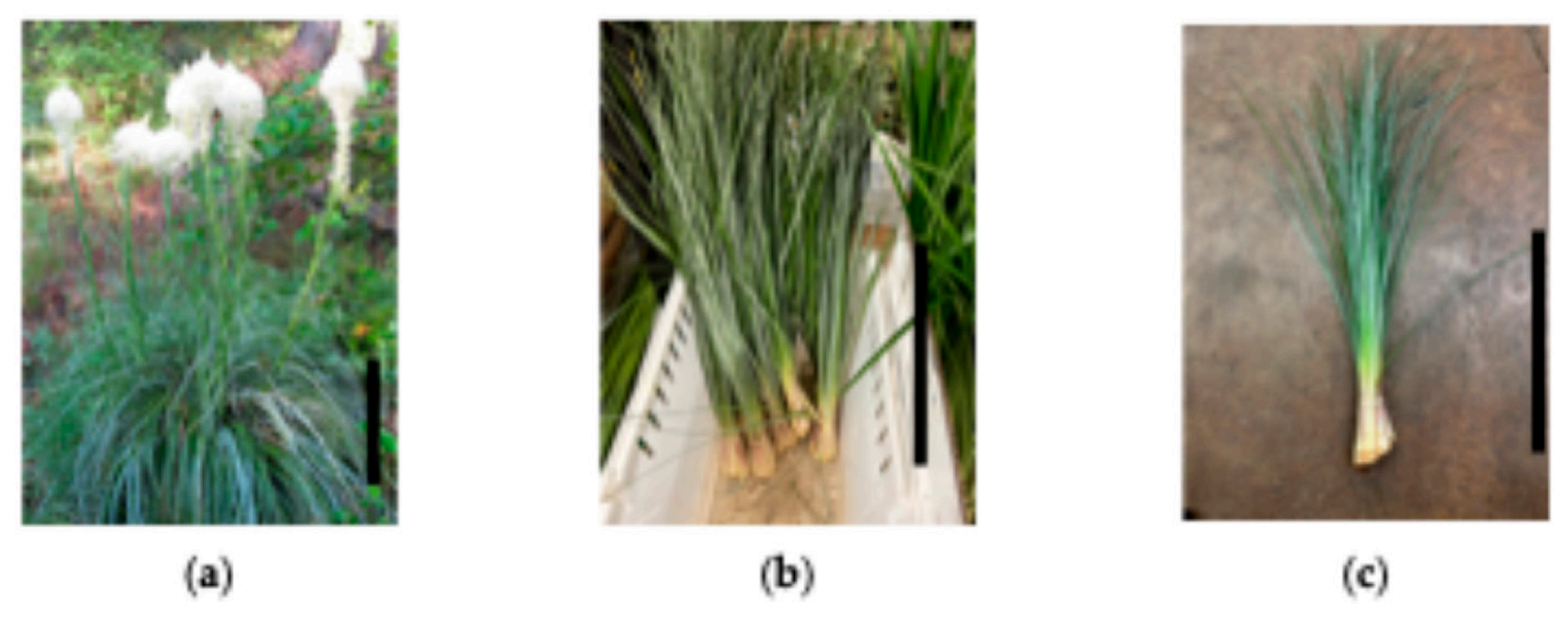
Bear grass, *Xerophyllum tenax*, as a commercial crop: (**a**) plant phenotype of a mature bear grass plant in the wild; most populations are wild-harvested, (**b**) example harvested bunches for the wholesale flower market, with approximately 50 leaves/bunch and (**c**) an individual bunch of leaves for use as a cut foliage for filler material in floral designs. Photo credits: (**a**) Barbara Mumblo, U.S. Department of Agriculture, Forest Service, https://www.fs.usda.gov/wildflowers/plant-of-the-week/xerophyllum_tenax.shtml, accessed on 1 March 2026, (**b**,**c**) Neil Anderson, courtesy of Koehler & Dramm Wholesale Florists, Minneapolis, MN. Scale bar = 25 cm.

One species within the family is a commercial crop, bear grass, *Xerophyllum tenax* (Pursh) Nutt. (=*Nolina macrocarpa* S. Watson) which is cultivated and/or wild harvested [19] for cut foliage (Figure 1a,b) and used in floral designing (Figure 1c) [20,21,22,23]. *Schoenocaulon* spp. have similar leaf structures (linear lanceolate), although bear grass leaves are much longer (30–100 cm × 2–6 mm, length × width), greener and more prolific.

Bear grass seeds are classified as having physiological dormancy (Majerus) and require 90–120 days of cold-moist stratification to germinate [24]. Commercial seed germination recommendations are 16 weeks of cold, moist chilling (stratification) at 3–5 °C for fresh seed [25]. Other reports, however, reported that no scarification or stratification was required for germination [26].

The flowers form spicate to racemose inflorescences [6,7,12]. *Schoenocaulon* gynoecia have a superior ovary (hypogynous) with free styles and simple stigmas [10,27]. Each ovary is composed of three septicidal carpels, containing 6–12 ovules per locule, which develop into 1–4 seeds per locule. The mature raceme produces seed in capsules which dehisce along 1–3 locule suture lines [28]. Architecturally, a mature (reproductive), established plant (>5 years old) has multiple inflorescences arising from a cluster of bulbs.

*Schoenocaulon* seed morphology includes a visible raphe (seam-like union) and a minute embryo embedded in a large endosperm. Seed germination in *S. officinale* is epigeal [10,27]. A well-established root system quickly develops in establishing seedlings (Years 1–2). At first flowering (~Years 1.5–3), a single inflorescence is produced per bulb. In subsequent years, multiple daughter bulbs will be produced from the original mother bulb, each of which will flower [29]. Thus, multiple inflorescences will be generated in each established flowering plant bulb cluster.

Breeding and domestication of *S. officinale* has commenced with phenomic, ionomic, and genomic characterization [4,30]. Crop relatives in the genus *Schoenocaulon* are of interest for study during the process of *S. officinale* domestication and could also serve as green pesticide crop alternatives.

*Schoenocaulon texanum* Scheele, Texas feathershank or Texas sabadilla, is the most northerly distributed taxon in the genus, native to central and western Texas and eastern New Mexico (USDA winter hardiness zones Z8a/b to Z7a) but also extends further south into Mexico (Z11) [6,7,10,28,31]. Its cold tolerance may increase the potential production range for sabadilla green pesticide production. As a diploid, geophytic species (2*n* = 2*x* = 16), it is most closely related to *S. dubium* (native to Florida and Georgia) [32,33], based on internal transcribed spacer (ITS) nuclear ribosomal DNA (nrDNA) ITS-1, 5.8S, and ITS-2 data [6,7].

**Figure 2:**
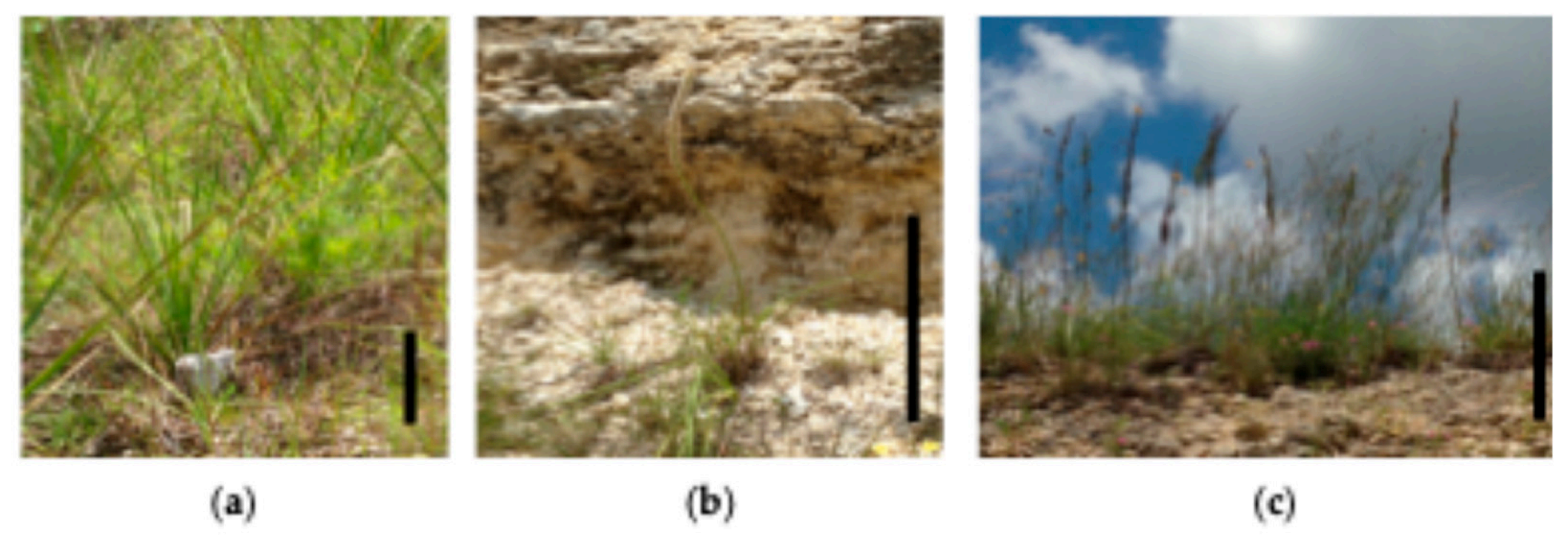
Wild, extant plant populations of *Schoenocaulon texanum* at various stages of plant development: (**a**) vegetative growth, Tarpley, TX, 9 June 2016; (**b**) flowering, Bandera, TX, 9 June 2016; (**c**) seed production, Winter, TX, 12 June 2016. Photo credits: Neil Anderson, Robert Suranyi. Scale bar = 25 cm.

*Schoenocaulon texanum* produces a tunicate bulb like *S. officinale* (2–4.5 × 1.2–4 cm dia.), growing in the chaparral in dry and rocky limestone areas with sparse topsoil [10,28]. Each established seedling bulb or “mother bulb” successively produces asexual clones or “daughter bulbs” in subsequent years of growth (Figure 2a), which occur in a circular pattern around the mother bulb’s basal plate [28,29]. Vegetative growth from each bulb produces 5–8 lanceolate evergreen leaves/year (reaching <60 cm in length and <6 mm in width; Figure 2a). Floral scapes range in height from 13.5 to 55 cm with up to 250 flowers/raceme, opening acropetally in spring (Figure 2b) to the beginning of summer or, occasionally, later in summer—early fall after “unseasonable rainfall” [28]. Annual rainfall differences also affect plant morphology, with lower rainfall resulting in “small stature” with short racemes and fewer flowers which may wither before maturation and immature ovaries with ~1 locule producing any seed (Figure 2c) [28]. Thus, seed production is variable between seasons and unpredictable, limiting seed testing.

Seed capsules have 1–3 locules, producing 2–8 seeds/locule, depending on pollination, levels of seasonal rainfall each year, or other abiotic factor(s) [28]. As with flowering, seed ripening occurs acropetally. Racemes turn brown-black in coloration and remain vertical for several years after seed dehiscence and flower stalk death (Figure 2c). Subsequent racemes are regenerated from each bulb in successive years.

*Schoenocaulon* seed morphology includes a visible raphe and a minute embryo embedded in a large endosperm. Seed germination in *S. officinale* is epigeal with a single cotyledon, since it is monocotyledonous [10,27]. Venezuelan populations of extant *S. officinale* seed had 97% viability in tetrazolium (triphenyl tetrazolium chloride or TTC) seed germination tests [9]. In the only replicated field study to date, germination of *S. officinale* ranged from 75% to 79% with no dormancy [10,27]. Seed viability standards for any *Schoenocaulon* spp. are nonexistent [34,35].

Seed germination standards are required for seed-propagated crops, establishing any postharvest treatments (e.g., scarification, after-ripening) or dormancy present that need to be circumvented (e.g., stratification) for fresh seed. Once the seed treatments have been administered for commercial seed lots, germination standards identify the best type of test (germination paper, soil), duration of the testing, expected number of weeks of germination post-sowing for the test duration (i.e., G1 = week 1 or day 7, etc.), expected % germination per week and final/adjusted final % germination [34,36]. Standard germination tests provide stand establishment levels of a seed lot planted under optimal conditions [37]. Adjusted final % germination values for ornamental, small-seeded crops range from 85–100% [34,36].

Long-term storage of seed may reduce % germination or viability as respiration continues even under orthodox seed storage conditions. Time, temperature, and relative humidity during storage of orthodox seed are critical factors affecting seed deterioration [34,35]. Additionally, low-quality seeds deteriorate more quickly than vigorous ones with a corresponding reduction in viability [38]. Since long-term storage over years is prohibitive, vigor tests are predictive of field emergence under a wide range of environmental conditions [37]. Accelerated aging is a routine vigor test that exposes seeds for a short time period (24–72 h.) to high temperatures (41 °C) and high relative humidity (75–100%), followed by standard germination tests [37,39]. In addition to being reproducible and economically feasible, vigor testing provides additional characterization of seed quality, uniformity and consistent ranking of seed lots [37]. Tekrony [40] found that accelerated aging tests correlated with field emergence and stand establishment, even though they are lower than laboratory germination tests [38]. The most important factors for rigorous vigor testing are oxygen, seed moisture and temperature [37].

Jian-hua and McDonald [41] proposed a new method of accelerated aging, i.e., saturated salt accelerated aging (SSAA), which provides a more accurate approach to test the small-seeded germination, in crops such as onion (*Allium cepa* L.), lettuce (*Lactuca sativa* L.), and rice (*Oryza sativa* L.) [34,37,39,42]. Vigor testing using SSAA in large-seeded crops, such as corn (*Zea mays* L.), wheat (*Triticum aestivum* L.), soybean (*Glycine max* Merr.) and sunflower (*Helianthus annuus* L.), has also proven successful [37,43]. The use of saline solutions (NaCl or MgCl) with lowered relative humidity incubation levels slows down water absorption as well as seed microflora (fungal) growth in SSAA tests [41].

Information is needed for early evaluation and domestication of a species. For *S. texanum*, significant knowledge gaps need to be addressed. Wild, extant collection of the taxon, coupled with phenotypic and chemotypic characterization of the populations, alkaloid content of the seeds, and soil conditions for future seed germination or production need assessment. We acknowledge that other biotic and abiotic factors could have an impact, but these are beyond the capacity for the current study. Seed germination levels (percent) of untreated *S. texanum* seed, as well as long-term viability, await discovery. The goal of this research was to quantify reproductive capacity for seed production traits, soil conditions, seed alkaloid content, and determine seed germination and vigor in extant, wild *S. texanum* populations of open-pollinated seed collected in the State of Texas, its native habitat. Research objectives and null hypotheses were to:

(a) Quantify reproductive capacity for traits associated with seed production, H_o_: There is no difference among and within populations and plant genotypes for the number of: (i) bulbs/plant, (ii) inflorescences/plant, (iii) seed capsules/plant, (iv) seeds/plant; seed dimensions (length/width) or 100 seed weight.
(b) Test field soil samples from population sites for differences in content (pedological environment conditions): H_o_: There is no difference among and within populations and plant genotypes for pH, organic matter, electrical conductivity or EC, and macro- and micro-nutrients.
(c) Determine alkaloid production in seed, H_o_: There is no difference among and within populations and plant genotypes for qualitative and quantitative alkaloid content.
(d) Determine standard seed germination levels in untreated seed, H_o_: There is no difference among and within populations and plant genotypes for percent seed germination across six germination weeks (G1–G6).
(e) Test the effects of accelerated aging tests on seed germination and viability changes at elevated temperatures, H_o_: There is no difference among and within populations and plant genotypes for percent accelerated aging seed germination across six germination weeks (G1–G6).

## 2. Results

### 2.1. Pedological Environment Conditions

Soil samples collected from the six population sites where plants for the tested seed lots were growing showed variation for all soil characteristics within and among the Spurway system, Exchangeable ion method and Microwave digest with HNO_3_ ICP (pH, organic matter, electrical conductivity or EC, macro- and micro-nutrients) analyses (Table 1). As would be expected with wild population soil sampling, there was also disparity of sampling sites’ results with the Spurway greenhouse soil standards (Table 1).

Organic matter levels in all soil samples, with a range of 1.9–7.1%, were lower than the Spurway standards (33–100%; Table 1). The pH values ranged from 7.8 to 8.2, all of which were lower than standard pH levels for greenhouse media (6.2–6.5). Electrical conductivity (EC) or relative dissolved soluble salt levels were non-saline, 0.2 mmhos/cm (millimhos per centimeter), for all soil samples.

The concentration of nitrate-nitrogen (NO_3_-N) from all samples ranged from 0.72 to 4.57 ppm, averaging 2.33 ppm, which was significantly lower than the Spurway standard (Table 1). NH_4_N levels were slightly lower than for NO_3_-N. Olsen P levels were 1–4 ppm, falling below the Spurway standard of 5–10 ppm. Bray P tests could not be performed since the soil pH > 7.4. Potassium levels, NH_4_OAc-K, averaged 85 ppm (Spurway) versus 157.84 ppm (exchangeable ion method) and were higher than the Spurway standards (Table 1). Calcium levels (exchangeable ion method) ranged from 4442.4 to 8028.9 ppm and were higher than Spurway standards. Micronutrient levels remained low for Fe, Mn, Zn, and Cu (Table 1). There were detectable levels of all heavy metals tested (Al, Cd, Cr, Ni, Pb). Based on these differences for all pedological analyses, the null hypothesis (H_o_) is rejected.

### 2.2. Alkaloid Analysis

The insecticidally active alkaloids, cevadine and veratridine, are found in the seeds of Sabadilla, *Schoenocaulon officinale*, but were undocumented for *S. texanum*. Bulk seed lots of three *S. texanum* populations (Table 2), in comparison with *S. officinale* as the standard, were quantified for alkaloid levels of cevadine (%) and veratridine (%) on a weight basis using standard extraction and high-pressure chromatography (HPLC) chemical analyses [8,9].

**Figure 3:**
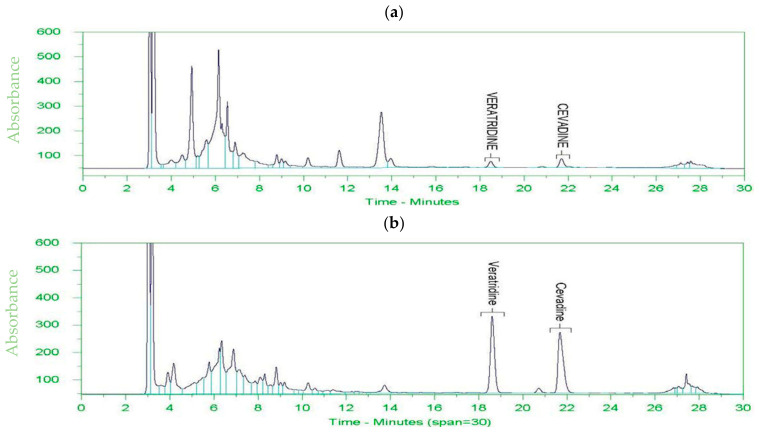
High-pressure liquid chromatography (HPLC) chromatograms or graphical outputs (absorbance) of de-oiled (**a**) *Schoenocaulon texanum* (St-1 seed lot) and (**b**) *S. officinale* (standard comparison) alkaloid extracts showing peaks representing the two alkaloids, veratridine (retention time~18.5 min) and cevadine (retention time~21.8 min). Impurities (unidentified compounds) eluted off at earlier retention times (3–16 min). Peak heights denote compound concentrations.

*Schoenocaulon texanum* seeds share most of the physical and biochemical characteristics of *S. officinale* (Table 2; Figure 3). Seed weights of *S. texanum* ranged from 3.3 mg (St-2) to 5.2 mg (St-1; Figure 3a) for the HPLC analysis, all slightly lower than *S. officinale*, 6.3 mg (Table 2; Figure 3b; *see also* Table 3 seed measurement data below). Seeds of *S. texanum* are 30% smaller than *S. officinale*, but similar in appearance, hardness, color and shape. Both species have similar oil contents by *w*/*w*%. The equilibrium moisture content of *S. texanum* is lower than that in *S. officinale* by about 25%, which would be an advantage in processing.

The percent cevadine content of *S. texanum* ranged from 0.03% (St-6) to 0.51% (St-1) and was also ~20% lower than the average content in *S. officinale* (2%; Table 2). There were higher concentrations and levels of impurities in *S. texanum* (Figure 3a, 3–16 min retention time) than *S. officinale* (Figure 3b). Percent veratridine levels were higher than cevadine, ranging from 0.27% (St-2) to 0.29% (St-1, St-6), although *S. texanum* was ~1/3 the levels in *S. officinale* (1.2%). Ratios of cevadine: veratridine (C:V) were similar across both species (1.6–1.8) except for St-6 (0.1; Table 2). Finally, the combined alkaloid levels varied widely within and among taxa, with *S. texanum* being 0.31–0.8% in contrast with *S. officinale* (3.6%). Thus, we reject the null hypothesis (H_o_) that there are no differences among and within species, populations, and genotype seed alkaloid levels.

### 2.3. Plant Growth and Reproductive Traits

#### 2.3.1. Bulb Production

The mean number of bulbs/clump ranged from two (St-3, St-7) to 12 (St-4; Table 3). A clump is defined as the group of mother bulb and clonal daughter bulbs of a single genotype that grow together in a clump configuration. There was no significant difference in mean bulb number/clump across populations and plants (*p* = 0.381). Thus, mean separations could not be performed and we fail to reject the null hypothesis (H_o_). There was a pooled average of 4.34 bulbs/plant clump (Table 3), ranging from one to 13 bulbs, demonstrating a range in age, with the youngest age at flowering having only 1 bulb/plant clump. The number of bulbs/clump was positively correlated with the mean number of flower stems/plant (*r* = 0.505), total number of seed capsules/plant (*r* = 0.224), the total number of seeds produced/plant (*r* = 0.447), and mean seed width (*r* = 0.214; Table 4), none of which were significant. Remaining trait correlations were negatively associated with bulb number. Most likely, the mother bulbs produce at least one daughter bulb each year. However, the length of the mother bulb’s life is unknown.

#### 2.3.2. Flower Production

On average, there were one to eight flower stems/plant in the wild populations, depending on the age of the original mother plant. Most bulbs had only 1 flower stem/plant, although occasional exceptions were found (St-1, St-9 with 2 flower stems/plant for one of the plants; Table 3). There was no significant difference in the number of flower stems/plant (*p* = 0.745). Thus, mean separations could not be performed and we failed to reject the null hypothesis (H_o_). This trait was positively correlated, although not significantly, with most of the seed production traits, ranging from *r* = −0.041 (mean seed width) to *r* = 0.556 (total number of seed capsules/plant; Table 4).

#### 2.3.3. Capsule and Seed Production

The 35 flowering inflorescences collected from ten bulb genotypes produced a total of 986 capsules (Table 3). Capsule production/inflorescence or bulb ranged from 32 (St-7) to 209 (St-1), with mean values of 15.33 (St-6) to 59 (St-5) and differed significantly (*p* < 0.001). Seed lot genotypes St-1, St-4 and St-5 were significantly higher in capsule production than all other seed lot genotypes but overlapped with St-8 and StG-1 (Table 3). Thus, we reject the null hypothesis (H_o_). Capsule production/inflorescence had a correlation of *r* = 0.072 (no. of flower stems/plant), whereas all other trait correlations were negative (Table 4).

A total yield of N = 3733 seeds was produced from the 35 flowering inflorescences, with a mean number of 373.3 seeds produced/plant (Table 3). The total number of seeds produced/plant ranged from 203 (St-5) to 657 (StG-1; Table 3) but was not significantly different among seed lots (*p* = 0.087). Thus, we fail to reject the null hypothesis (H_o_). The total number of seeds produced/plant was positively, but not significantly, correlated with the number of bulbs/clump (*r* = 0.477), mean number of flower stems/plant (*r* = 0.545), total number of seed capsules/plant (*r* = 0.682), total number of seeds/inflorescence (*r* = 0.053), mean number of seeds/capsule (*r* = 0.005), mean 100 seed weight (*r* = 0.166) and mean seed width (*r* = 0.17; Table 4). All other traits were negatively correlated with the total number of seeds produced/plant.

Total number of seeds/capsule within each inflorescence ranged from 1 to 12, with most capsules bifurcating into two seeds each. Mean number of seeds/capsule ranged from 1.96 (St-1) to 7.59 (St-6; Table 3). The pooled mean seed production/capsule was 4.56 (Table 3). Despite the wide range in variation, however, there was no difference among seed lots (*p* = 0.129) and we failed to reject the null hypothesis (H_o_). As would be expected, there was a significant, positive correlation (*r* = 0.699) between the mean number of seeds/capsule and the mean number of seeds/inflorescence. All other trait correlations were slightly, but insignificantly, negatively or positively correlated.

There was no significant difference in mean 100 seed weight among seed lots (*p* = 0.257), although the range was 0.23 g (St-8) to 0.55 g (St-1, St-6; Table 3). Thus, mean separations were not feasible and we failed to reject the null hypothesis (H_o_). The mean 100 seed weight data pooled across seed lots = 0.45 g, with a pooled mean individual seed weight = 0.0045g/seed (Table 3). Mean 100 seed weights were not significantly correlated with any other phenotypic trait, ranging from *r* = −0.426 (mean number of seeds/capsule) to *r* = 0.515 (mean number of flower stems/plant; Table 3).

Mean seed lengths were very highly significantly different among seed lots (*p* = 0.005) and we, thereby, reject the null hypothesis (H_o_). Seed lot StG-1 had the significantly lowest seed length (3.55 mm) versus the significantly highest of 4.95 mm (St-8) and 5.4 mm (St-9; Table 3). Mean seed widths were also very highly significantly different among seed lots (*p* = 0.005). Seed lot St-1 had significantly lower mean seed width (0.49 mm) than all other seed lots, with the highest =1.3 mm (StG-1). As would be expected, mean seed length: width ratios were also very highly significantly different (*p* = 0.005), differing from the lowest l:w = 2.73 mm, 2.96 mm, 3.07 mm (St-G-1, St-6, St-4, respectively) which were significantly different than l:w = 4.69 mm, 4.78 mm, 4.95 mm, 5.14 mm (St-9, St-3, St-8, St-2, respectively; Table 3). The highest l:w ratios occurred with St-1 (9.82 mm). All other mean l:w ratios overlapped with the lowest ratios.

### 2.4. Standard Seed Germination Tests

The number of seeds/g ranged from 182 g (St-1) and 183 g (St-6) to the highest of 440 g (St-8), with a pooled average of 268 g (Table 5). The range in variation would be expected since the seed length, width, and l:w ratios were significantly different, as presented above (Table 4). Seed weights in and out were both significantly different among seed lots (*p* ≤ 0.001; Table 5) and we reject the null hypothesis (H_o_). As would be expected, weights in and out were highly and significantly correlated, *r* = 0.99 (Table 6). The weight difference, however, was not significantly different for seed lots, with a pooled average of 0.06 g (Table 5). Weight differences were not correlated with weight in (*r* = 0.35) but were significantly correlated with weights out (*r* = 0.45; Table 6).

None (0%) of the seeds germinated in the first two weeks (G1, G2) of the germination test. Seed germination commenced in G3 (week 3). Percent germination in G3 was very highly significantly different among seed lots (*p* ≤ 0.001) with a wide range in variation, from the significantly lowest of 7% (St-7) to the significantly highest at 77% (St-4; Table 5). Seed lot St-6 was not significantly different than St-4, with 56% germination in G3. The G3% germination was significantly correlated with weight in (*r* = 0.45) but more so with weight out (*r* = 0.59). It was not significantly correlated with the weight difference (*r* = 0.44; Table 6), however.

**Figure 4:**
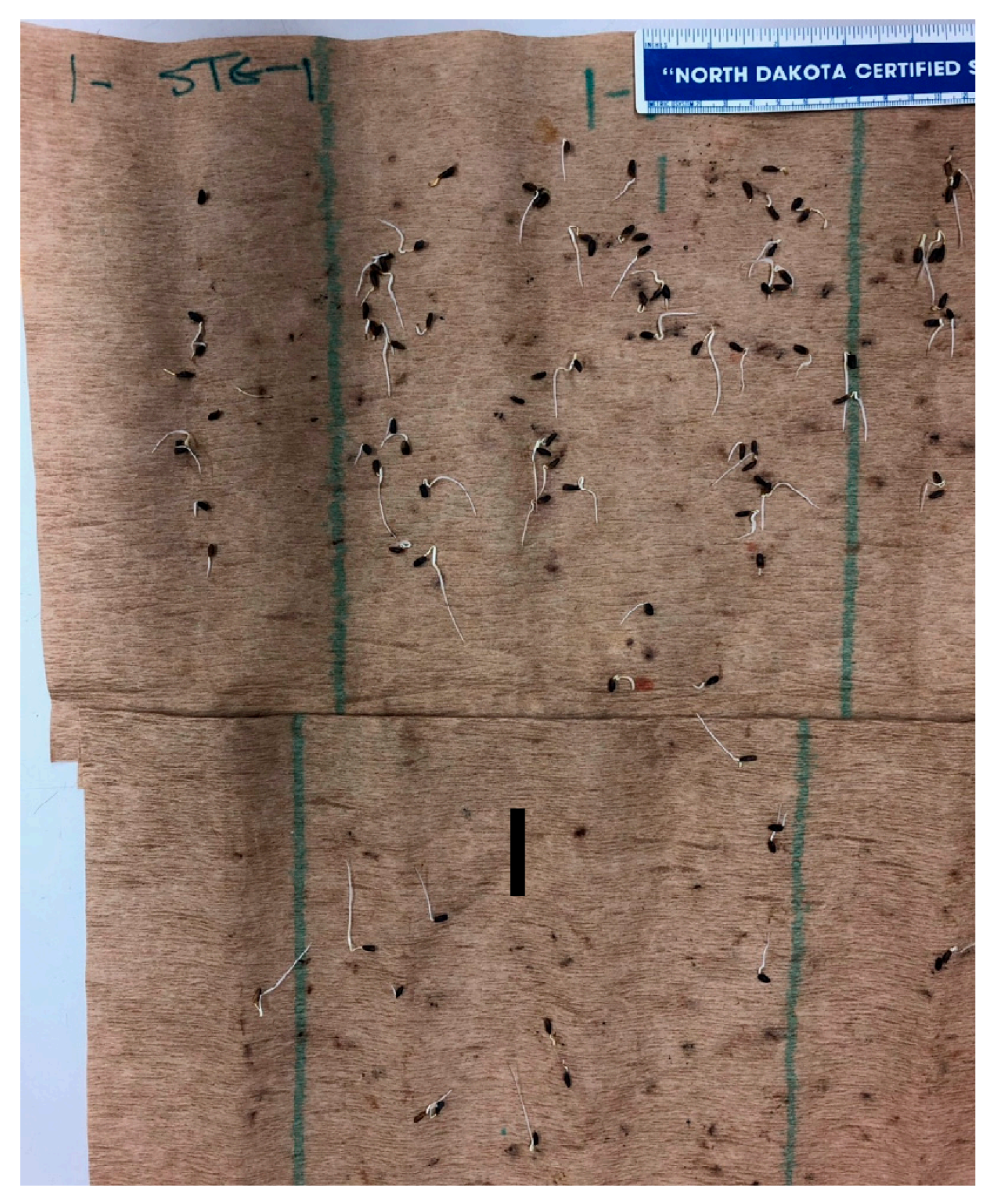
Example standard seed germination test results (G4, germination week four) for one germination paper replication (n = 100 seeds) of *Schoenocaulon texanum* wild population seed lot, StG-1. Photo credit: Michele Schermann. Scale bar = 50 mm.

Week 4 (G4) seed germination was highly significantly different by seed lots (*p* ≤ 0.01). By G4, significant quantities of seed had germinated which necessitated increased time to assess germination (Figure 4). Seed germination ranged from 4 (St-5, St-7) which was significantly lower than the highest with 25% in G4 (St-1; Table 5). All other seed lot % germination in G4 overlapped with either of these two statistical groupings. The G4% germination was significantly correlated with weights in (*r* = 0.62) and out (*r* = 0.57; Table 6). It was not significantly correlated with either the weight difference (*r* = −0.10) or G3% germination (*r* = 0.18).

By G5, seed germination had dropped such that % germination did not differ significantly (*p* = 0.448) among seed lots. Thus, mean separations could not be performed. The pooled mean % germination in G5 was 3.2% (Table 5). Percent germination in G5 ranged from 2% (St-9) to 28% (StG-1). The G5% germination was positively correlated with all traits, although the *r* values were low, but only significantly correlated with G4% germination (*r* = 0.43; Table 6).

In G6, % germination differed significantly among seed lots (*p* = 0.021). The significantly lowest was 1% (St-3, St-4) to the highest of 5% (StG-1; Table 5). The G6% germination was only positively and significantly correlated with later germination weeks, G4 (r = 0.69) and G5% seed germination (*r* = 0.43; Table 6), but not earlier weeks (G3, for example). In fact, the latter correlation was negative but not significant (*r* = −0.01).

The adjusted final % germination averaged for all seed lots was 55%; seed lots differed significantly (*p* ≤ 0.001). Thus, a significant range occurred among seed lots from a low of 14% (St-7) to the highest at 91% (St-4; Table 5), with nearly all seed lot means being significantly different than the others. Correlations of adjusted final % germination were significantly and positively correlated with four traits: weight in (*r* = 0.74), weight out (*r* = 0.74), G3% germination (r = 0.89) and G4% germination (*r* = 0.59; Table 6). These four traits—over all other traits—significantly impact the adjusted final % germination. All other trait correlations with the adjusted final % germination were positive but not significantly correlated.

A total of four seed lots or 40% of tested samples had ≥70% adjusted final % germination (St-1, St-4, St-6, StG-1; Table 5). These and, especially the St-4 population, would be definitive sources for high seed germination.

### 2.5. Saturated Salt Accelerated Aging Seed Germination Tests

**Figure 5:**
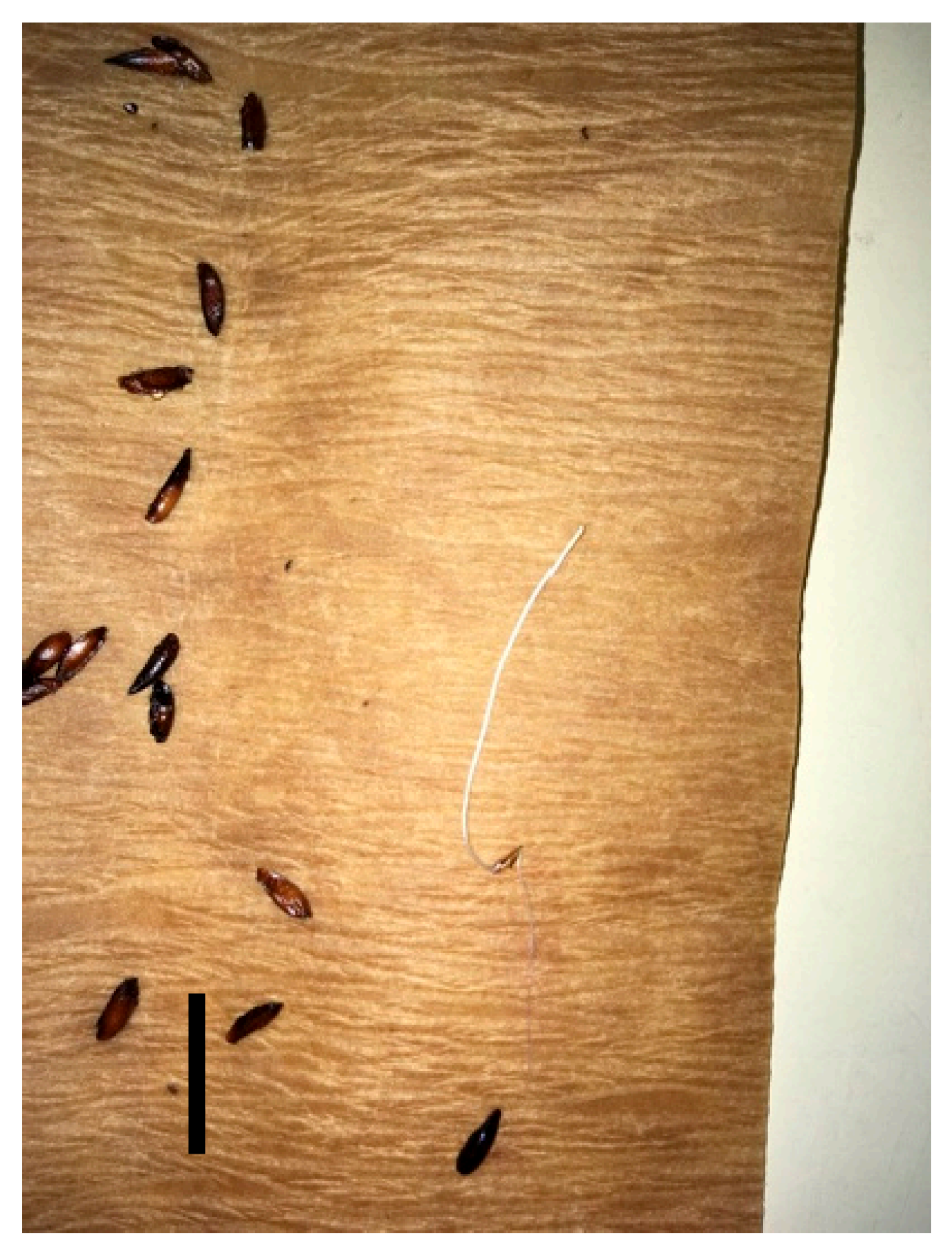
Example saturated salt accelerated aging seed germination test of *Schoenocaulon texanum* seed lot collected from a wild, extant population. Note the darkened seed coats and one germinated seedling. Photo credit: Michele Schermann. Scale bar = 50 mm.

In the SSAA tests, there was no seed germination (0%) in weeks G1–G2 for any of the seed lots, which was the same as standard germination tests. The SSAA seed germination also commenced in week G3 (Figure 5, Table 7), again mirroring the results for standard germination (Table 5). There were significant differences among seed lots in all weeks for % germination; the entire set of G3–G6% germination ANOVAs had *p* ≤ 0.001. Thus, we reject the null hypothesis (H_o_).

Week G3 SSAA mean % germination ranged from 6.5% (St-7) to 76.5% (St-4; Table 7). Most G3 means were significantly different among seed lots (*p* ≤ 0.001). Correlations of G3% germination with the other weeks (G4, G5, G6) were nearly zero (positive or negative *r* values; Table 8) and were not significantly different. However, the G3% germination had the greatest positive and significant effect on the SSAA-adjusted final % germination (*r* = 0.93, *p* ≤ 0.01). Even though accelerated aging G3% germination was slightly lower than most G3 standard % germination, the statistical comparison of SSAA with standard germination tests for G3 did not differ significantly (*p* = 0.893). However, seed lots remained significantly different (*p* ≤ 0.001).

Mean % SSAA germination in week G4 ranged from 3.5–4.0% (St-7, St-5, respectively; Table 7), which were significantly lower than all other seed lot means, with the significantly highest at 25.0% (St-1). Correlations of the G4% germination SSAA were significantly and positively correlated for all subsequent germination weeks, i.e., G5 (*r* = 0.78) and G6 (*r* = 0.82; Table 8). The adjusted final % germination for SSAA was significantly and positively correlated with G4 (*r* = 0.61, *p* ≤ 0.01; Table 8). Thus, G3 and G4 were the only two germination weeks positively and significantly correlated with the adjusted final % germination for SSAA. While several accelerated aging % germination in G4 were slightly lower than corollary G4 standard % germination, the statistical comparison of both tests in week G4 did not differ significantly (*p* = 0.853). Seed lots, however, were significantly different (*p* ≤ 0.001).

Week G5 SSAA % germination dropped precipitously from weeks G3 and G4 levels, ranging from 1.0% (St-3, St-9) to 8.5% (St-1; Table 7). The G5% germination SSAA were significantly and positively correlated with the prior weeks’ (G4, *r* = 0.78) and subsequent week (G6, *r* = 0.74; Table 8). Similar to weeks G3 and G4, the statistical comparison of SSAA and standard germination tests for week G5 was not significant (*p* = 0.91). However, while seed lots were still significantly different, the *p*-value (*p* = 0.002) was less significant than for weeks G3 and G4. As a result, only seed lot St-1 was significantly higher than all other seed lots. Thus, SSAA gradually reduces the genetic seed lot or population differences.

Mean % germination SSAA in week G6 had the lowest range in distribution, 0.5% (St-3) to 3.5% (St-1; Table 7), as would be expected while germination progressed and aging duration increased. As noted for week G5, the week G6 SSAA % germination was only significantly and positively correlated with weeks G4 and G5, but not any other weeks nor for the adjusted final % germination (Table 8). Statistical comparison of SSAA and standard germination tests in week G6 was not significant (*p* = 0.862). Seed lots were less significantly different (*p* = 0.01) in week G6, resulting in seed lots St-3 and St-4 being significantly lower than St-1, while all other seed lots overlapped.

The pooled (weeks G3–G6) adjusted final % germination of SSAA seed lots differed significantly (*p* ≤ 0.001). Pooled values ranged from 12% (St-7) to 90% (St-4, Table 7). Three seed lots had >70% adjusted final % germination levels: St-1, St-4, and St-6 (Table 7). Comparison of SSAA and standard adjusted final % germination was significantly different for both the treatments (*p* = 0.001) and seed lots (*p* ≤ 0.001).

## 3. Discussion

### 3.1. Pedagogical Environmental Condition

Soil characteristics varied among the population sites tested (Table 1). Organic matter levels were very low, which is typical of the well-drained, dry, sandy, rocky, Caliche (with high levels of soluble calcium carbonate) soil type found in the Texas hill country landscapes [44,45]. Similar organic matter was found in wild populations of *S. officinale* in Venezuela [46] and Honduras [27]. Characteristic high pH levels (7.8–8.2) of alkali soils exceeded expectations for Spurway levels of greenhouse crops (6.2–6.5). These were higher than soil tests of *S. officinale*, pH = 6.1–6.2, tested in La Soledad Experimental Station, Comayagua, Honduras [27]. There were surprisingly low EC levels, as well as NO_3_N, NH_4_N, P, K, Fe, Mn, Zn, and Cu among all population locations (Table 1). However, high levels of Ca occurred throughout due to the Caliche soil type with characteristically high levels of CaCO_3_ [45]. Detectable levels of heavy metals (Al, Cd, Cr, Ni, Pb) were also common at all sites. Soil tests for *S. officinale* sites in Honduras showed significantly higher levels of macro- and micro-nutrients [27] than those found in *S. texanum* soils in Texas.

For the species to be bred and domesticated as a green pesticide crop for alkaloid production, significant trialing would be required to assure consistent, stable performance and high seed yield. The anticipated field soil conditions would mimic most of these soil test levels, although higher N levels and irrigation would encourage plant growth as discovered in commercial production of *S. officinale* [4,30]. Soil recommendations for cultivation of *S. texanum* could be patterned after bear grass production: well-drained, sandy-peat soil types [26]. Additional *Schoenocaulon drummondii* Gray soil recommendations are a range of soil types (sandy, loam, clay) with high organic matter and adequate drainage [46,47]. The presence of heavy metals/metalloids in the soil would be undesirable in a green pesticide crop [4]. Current molecular technologies, such as gene editing, have been harnessed to create transgenic plants or microbes to alter heavy metals into less toxic forms via crystallization or phytomineralization to sustainably detoxify soils [48]. Further testing would be required to determine the best soil type(s) to maximize establishment, growth, and seed yield as a green pesticide crop.

### 3.2. Alkaloids

*Schoenocaulon texanum* seeds share physiological and biochemical seed traits with *S. officinale*, although genetic variation was evident (Figure 3). Both species share seed traits, especially appearance, seed coat hardness, color, and shape. However, in the *S. texanum* seed lots and populations tested in the HPLC analyses [8], a ~30% seed weight reduction was found. Seeds from *S. texanum* (4.5 mg) are slightly lighter than those of *S. officinale* (6.3 mg), although these differences could be due to environmental rather than genetic (species) effects which could impact alkaloid production levels. The caveat that the *S. texanum* seeds tested were collected from wild, extant populations could significantly affect seed weights. Future assessments in commercial production would be required to determine potential shifts in seed weights and allow for selection of superior genotypes tested.

An unexpected finding within *S. texanum* seed lots was a decrease in equilibrium moisture levels, i.e., ~25% lower than *S. officinale*. This trait would have a significant advantage in commercial seed processing prior to alkaloid extractions.

Both of the *Schoenocaulon* species tested contain alkaloids, which are amines derived from NH_4_ [11]. Cevadine levels among *S. texanum* populations (0.03–2.51%) were lower than in *S. officinale* (2.0%). There were corresponding decreased veratridine levels in *S. texanum* (0.27–0.29%) compared with *S. officinale* (1.2%). Higher levels of total alkaloids in *S. officinale* have been reported, i.e., 3–6% [32] and 2.86–3.4% [30]. The combined alkaloid levels of *S. texanum* (0.31–0.8%) were significantly lower than those of *S. officinale* (3.6%). Intriguingly, the C:V ratios were the same for both *S. texanum* and *S. officinale*, with the notable exception of seed lot St-6. While these data might deter domestication of *S. texanum* for green pesticide (alkaloid) production, it must be noted that these are random population samples of seed lots from wild, natural conditions completely lacking in supplemental fertilization and irrigation application—both of which could significantly increase either cevadine and/or veratridine concentrations. For example, in *S. officinale* production studies in Mafinga, Tanzania, the precipitation concentration index (PCI) had significantly greater % total alkaloids in years with lower PCI [30].

### 3.3. Plant Growth and Reproductive Traits

#### Bulb Production

The wide range in *S. texanum* bulb number/clump (2–12) indicates varying age differences [49] among the genotypes sampled (Table 3). The total bulb number/clump has been termed “cluster” and the precise range in bulbs/clump (2–12) was also reported in wild populations of *S. officinale* [46]. Radloff and Anderson [30] report similar levels of bulb number/clump for *S. officinale*, increasing with age (years) in established clumps. There were no significant differences among *S. texanum* populations for bulb number/clump. Daughter bulbs remain connected to the mother bulb basal plate in other *Schoenocaulon* species, e.g., *S. officinale* [46], although these are not rhizomes [50] since they lack nodes. Since daughter bulb (agamic, clonal, or asexual) propagation from the basal plate increases the bulb number/clump, future studies on the rate of daughter bulb generation from *S. texanum* mother bulbs would provide selection data for domestication.

*Schoenocaulon texanum* bulb number was positively but not significantly correlated with the number of flower stems/plant, total number of seed capsules, total number of seeds produced/plant, and mean seed width (Table 4). While we did not measure bulb size, Emms [51] reported that larger, older bulbs of the related species, *Zigadenus paniculatus*, produced taller inflorescences with higher numbers of flowers/raceme (<250). The high bulb number/clump in *S. texanum* was unexpected, given the native habitat of rocky limestone crags. During the collection expeditions, we frequently needed to use deep-penetrating tools to extirpate bulbs from 0.25 m, since they were deeply rooted into the rock due to the action of contractile roots. Previous collections of *S. officinale* reported the need for augers or pick axes to extract bulbs and obtain soil samples [46]. Future research would be needed to determine the effects on commercial bulb production in open fields without the added soil pressures elicited in rocky soils.

### 3.4. Flower, Capsule, and Seed Production

Due to strong apical dominance, the production of one inflorescence/bulb in *S. texanum* is expected, similar to *S. officinale* [46]. These data are comparable with other tunicate bulb crops such as ornamental tulips (*Tulipa gesneriana*), daffodils (*Narcissus pseudonarcissus*) or hyacinth (*Hyacinthus orientalis*), as well as edible onion (*Allium cepa*) and green pesticide crops (*S. officinale, Zigadenus paniculatus*) [25,46,51]. Occasional occurrence of two inflorescences/bulb indicates a slight potential to increase this number, although the cause(s) of this are unknown; it has been theorized that removal or damage to the apical meristem early in flower bud development could be the causal factor [46].

Capsule production of *S. texanum* had a wide range in variation (32–209/inflorescence; Table 3). The St-1, St-4, St-5, St-8, and StG-1 seed lots were derived from plants producing the highest number of capsules. Such genotypes could be hybridized to potentially increase capsule and seed production as well as study heritability. Variation in capsule production may be due to either genetic differences and/or confounded environmental conditions of higher temperatures, drought, insect feeding, or other unknown causes. Flower bud abortion or lack of complete flowering occurs in wild *S. officinale* [46], which could significantly reduce capsule production. Future production research in common gardens would minimize these factors to singulate genetic effects.

The wide range in seed number/capsule (1–12) demonstrates high levels of variability in reproductive effort in *S. texanum*. The range in number of seed/capsule found in *S. texanum* is similar to that of related *Zigadenus paniculatus*, with a range of 6–14 [51]. These differences could be due to ovule number differences, pollination rates, as well as environmental effects that cause ovule and embryo abortion. Since *S. texanum* St-6 had the highest mean number of seed/capsule, coupling parental selection of this genotype with other genotypes with high seed values (St-1) could enhance seed production.

### 3.5. Standard Seed Germination

The earliest time at which *S. texanum* seed germination occurs under standard test conditions is three weeks after sowing (G3; Table 5). This matches previous reports for *S. officinale* seed germination [27]. The standard seed germination temperature tested (20 °C), a primary abiotic regulator of seed germination [52], was within the optimal temperature range (14–20 °C) reported in other geophytes with tunicate bulbs such as *Zephyranthes tubispatha* (Amaryllidaceae) [52] and *Trillium camschatcense* (Melanthiaceae) [53], as well as matching the germination temperature (20 °C) used for *Schoenocaulon officinale* [9]. Related geophytes with cold stratification requirements to break dormancy germinate at lower temperatures, e.g., *Zigadenus leimanthoides* and *Z. densus* (Melanthiaceae) [54].

Epigeal germination of this orthodox seed is the norm in this monocotyledonous genus [27]. The duration of the standard seed germination test period continued for another three weeks to G6 (Table 5). While we tested a small sample of *S. texanum* seed with TTC and found viable embryos, there were insufficient quantities of seed in any remnant seed lots to permit robust experimentation for either pre- or post-germination (>wk. 6) dormancy. Whether a percentage of the seeds were dead before or after germination testing or if they possess conditional/morphophysiological dormancy triggered by specific temperature or light regimes is unknown. However, since <100% germination occurred in all tested seed lots (in both standard and SSAA tests), the non-germinating seeds at the end of week G6 might subsequently germinate. Storage or dry after-ripening conditions in the seed vault could have impacted this. Future studies would be required to determine whether remnant, ungerminated seed (>G6) are still alive.

Venezuelan wild-harvested *S. officinale* seed was found to have 97% viability in TTC seed germination tests; no dormancy existed [9]. The *S. texanum* population St-4 would be closest to these findings for *S. officinale* [9,10,27]. It would be expected that additional germination might occur after week G6, as is characteristic in wild-sourced seed of undomesticated herbaceous perennials. Thus, we cannot say definitively that there is no evidence of dormancy in *S. texanum* seeds, unlike that of related *Xerophyllum tenax*, bear grass. Some populations of *Xerophyllum* have physiological dormancy, requiring 16 weeks cold-moist stratification at 3–5 °C for fresh seed [24,25]. Commercial seed germination recommendations for bear grass are 16 weeks (2688 h) of cold, moist chilling (stratification) at 3–5 °C [25]. Neither scarification nor stratification was needed for *S. texanum* germination.

There were significant differences in G3% seed germination at 20 °C among *S. texanum* seed lots. A wide range of expression, from 7% (St-7) to 77% (St-4; Table 5), indicates differential responses of populations and genotypes in each seed lot. The low-germinating lots may have slight dormancy, or they may need different germination conditions than we tested, although limited seed availability limited including additional environments.

Week G4 seed germination of *S. texanum* was high and significantly different among seed lots, although the levels were reduced significantly from G3. The highest standard seed germination in G4 was 25% (St-1). Ranked differences among weeks G3 and G4 were found, since the significantly highest levels of seed germination included St-4 in G3 but switched to St-1 in G4. Similar results occurred in weeks G5 and G6. Thus, adjusted final % seed germination is a much more inclusive indicator of seed germination potential.

Week G5 was the only week in which there were no significant differences in % seed germination among weeks. This may be due to lower levels and variation of seed germination in G5. Seed lot St-1 had the highest % seed germination in week G6 (5%).

Conducting standard germination tests for high-value and high-quality seed-propagated, green pesticide crops under optimal conditions maximizes efficiency in precision seeding and plug production to cost-effectively produce optimal levels of seedlings [55,56]. Adjusted final, pooled % seed germination was 55% and was correlated with seed weights in/out, as well as G3, G4% germination. Larger seed sizes contain greater carbohydrate and lipid reserves supporting longevity during seed storage and radicle emergence [35,41,54,56], even though smaller seeds (lower seed weights) absorb water faster than large seeds, particularly during AA, due to greater surface area to volume ratios [57].

Overall, seed lot St-4 (90.5%) had the highest standard % seed germination and was similar to one *S. officinale* seed lot (92%) [27]. As such, since the standard germination test is conducted in optimal conditions, these are the maximum female parent ability for a seed lot [57]. Similar maximum germination rates of 90% at 7–20 °C were reported in wild-collected seed of the geophyte, *Zephyranthes tubispatha* [52]. Additional *S. texanum* seed lots with high levels (>70% adjusted final germination) included St-1, St-4, St-6, and StG-1. These would be ideal parents to serve as a base for increasing seed germination levels to acceptable market standards. Thus, based on the present study, an initial germination standard for open-pollinated seed lots of *S. texanum* would be ≥70%. Germination standards are based on total or adjusted final % germination of a seed lot under ideal test conditions, rather than individual germination week percentages [35,37,39]. This germination standard overestimates yield potential or field establishment [57].

The *S. texanum* St-4% seed germination was similar to the only replicated field study of *S. officinale* seed germination (75–79%) [10,27]. Sexual propagation (seeds) in geophytes with long juvenility periods and time to flowering, while not as quick to generate flowering plants as asexual means, allows for wider genetic variation in crop production [52].

Four seed lots (40%) of tested samples had ≥70% adjusted final % germination (St-1, St-4, St-6, StG-1; Table 5). These and, especially, the St-4 population (90.5% germination) would be definitive sources for high seed germination. We would establish initial seed germination standards of *S. texanum* at 70% germination (20 °C, 4-week test) with the expectation of increasing this to ≥90% with breeding and selection of superior lines.

Plant breeders and seed physiologists could preferentially select to maximize seed lot germination in week G4, since the 2-week period of G3–G4 had the highest rate (%) of seed germination, which quickly diminished in weeks G5–G6. Potentially, breeding and selection for earlier *S. texanum* seed germination to occur in weeks G1–G2 would more closely match seed germination standards in flowering ornamental bedding plant (annual and perennial life histories) and herb/vegetable crops [34,36].

### 3.6. Saturated Salt Accelerated Aging (SSAA) Seed Germination

Vigor tests are critical for high-value seeds, such as green pesticide and ornamental crops, when germinated plug trays from pre-finisher growers require 100% germination and complete fill of plug trays as well as produce a vigorous, viable plant in the field [55,57]. Accelerated aging (41–43 °C, 100% relative humidity, RH, for ≤72 h.) is an established vigor test for small- and large-seeded crops [39,40,55,58]. Since AA increases seed moisture quickly and encourages fungal infections, the SSAA was developed to control the RH of the air with saturated salts [41]. Numerous research labs have confirmed the validity and predictability of the SSAA test to cause aging to correlate with longevity in seed storage and emergence for direct-sown field crops [39,40], as well as for early emergence and yield potential in glasshouse plug production [36,55]. The SSAA tests of *S. texanum* seed lots matched the standard seed germination tests in terms of timing for the commencement of seed germination (G3), the highest % germination in weeks G3-G4, and corollary decreases during weeks G5-G6 (Table 7). While increased respiration with higher temperatures in the SSAA tests could have, theoretically, delayed the start of seed germination beyond week G3, it did not occur.

Week G3 accelerated aging % seed germination reached a high of 76.5% in seed lot St-4 which was not significantly different from the seed lot G3 standard seed germination of 77% (Table 7). The significance levels did decrease with the SSAA, in comparison with standard germination which has been previously reported [55]. In weeks G3, G4, G5 and G6, there were no significant differences among seed lots for SSAA and standard germination tests. These findings match previous comparisons of SSAA and standard germination of viola (*Viola tricolor* L.), an ornamental bedding plant [55]. Week G3 accelerated aging % seed germination levels were correlated with the adjusted final % germination. Previous research with seed germination of the geophyte *Zephyranthes tubispatha* at elevated temperatures of 28 and 33 °C for durations of 3, 5, and 25 days had decreased % germination, although these were thermoinhibitory (high temperature) treatments rather than SSAA, followed by standard germination temperatures [52]. Likewise, accelerated aging in rice (*Oryza sativa* L.) significantly decreased seed germination as much as 75% [59].

In week G4, the SSAA % seed germination maxima was 25% for seed lot St-1. Week G4 data were correlated with both G5 and G6 as well as the adjusted final % germination in the SSAA tests. The highest SSAA % seed germination in week G5 was 8.5% (St-1), a considerable decrease from the previous two weeks (G3, G4). Week G5 germination was correlated with weeks G4 and G6. By week G6, the highest SSAA % seed germination equaled 3.5% in seed lot St-1. The precipitous decline in weeks G4-G6 SSAA % seed germination indicates that long-term storage of this seed would not be advisable, although the tested SSAA temperatures and duration do not translate directly into specific seed storage durations at specific temperatures [56,57]. This would require substantial seed lot sizes to determine specific seed storage and longevity recommendations.

Seed lot St-1 has a greater ability to withstand seed aging over time and maintain the highest level of seed germination. There may be specific environmental conditions in Bandera County, Texas, where St-1 originated that have selected for increased seed longevity. Likewise, the genetic makeup of this population may also be a causal factor. Molecular analyses, such as genotype by sequencing to generate low- or high-density single nucleotide polymorphisms (SNPs) coupled with genome-wide association studies (GWAS) and cell-type-specific transcriptomics [60] might be able to identify causal genes and alleles.

Adjusted final % seed germination in the SSAA tests was significantly different among seed lots, ranging from 12% (St-7) to 90% (St-4; Table 7). As found with standard % seed germination, seed lots St-1, St-4, and St-6 had the highest SSAA-adjusted final % seed germination. Thus, the SSAA vigor test standards for *S. texanum* would be ≥70%. The SSAA tests are particularly important for microbiotic or short-lived seeds [61]. It is unknown whether *S. texanum* seeds are microbiotic (1–3 years storage) or can survive longer (mesobiotic, macrobiotic). The SSAA vigor tests provide guidance for seed inventory management and anticipated longevity in long-term seed storage, as well as to establish fair pricing, determine carry-over of seed lots from year-to-year, and factors of plant growth requirements from germination to harvest [57]. When sufficient seed quantities are available, future vigor testing of this species could be conducted to determine electrical conductivity, lipid peroxidation, and unsaturated fatty acid content [59].

## 4. Materials and Methods

### 4.1. Germplasm Collection for Study

Germplasm for testing was from extant, wild-collected, open-pollinated (OP) populations of *S. texanum* from a plant collection trip to Texas, U.S.A., conducted by two authors (N. Anderson, R. Suranyi) in 2016. The OP seed was collected from a total of n = 10 genotypes from five non-sympatric, extant populations (Table 9).

### 4.2. Morphological, Chemical, and Soil Profile Data

In situ data collection on the plants during the collection trip included the number of bulbs/plant and the number of inflorescences/plant. Soil samples (n = 1 rep./site) were collected in six population sites for testing (Table 9) as follows. Each soil sample was collected at the base of the first plant observed in each population, with a 250 g sample collected as topsoil subtending the existing plant material. Samples were returned to the lab in resealable plastic bags (1.75 mil, 1 Quart Get Reddi^®^ Reclosable Food Service Bags, https://www.usplastic.com/catalog/item.aspx?itemid=128308&catid (accessed on 1 March 2026)) and kept at 3–5 °C until tested using (a) Spurway Greenhouse, Florist, & Nursery Crops, (b) Exchangeable ion method and (c) Microwave digest with HNO_3_ ICP analyses for field sample testing at the University of Minnesota, Department of Soil, Water and Climate’s Soil Testing Laboratory (http://soiltest.cfans.umn.edu/, accessed on 1 March 2026; Soil Job #181, 2015–16). Soil samples were evaluated for percent (%) organic matter or LOI OM, water pH, 1:1 electrical conductivity or EC (mmhos/cm), NO_3_−N (ppm or mg/kg soil), NH_4_-N (ppm), Bray P (ppm), Olsen P (ppm), NH_4_OAc-K (ppm), NH_4_OAc-Ca (ppm), NH_4_OAc-Mg (ppm), DTPA-Fe (ppm), DTPA Mn (ppm), DTPA Zn (ppm), DTPA Cu (ppm), SO_4_−S (ppm), exchangeable NH_4_OAc-K (ppm), exchangeable NH_4_OAc-Ca (ppm), exchangeable NH_4_OAc-Mg (ppm), exchangeable NH_4_OAc-Na (ppm), and hot water boron (ppm). The NH_4_OAc-Na (ppm) determined salt concentrations, rather than just EC values, since ECs represent dissolved solutes, including Na.

Alkaloid testing to determine two principal sabadilla ester alkaloids, percent *w*/*w* cevadine, veratridine, and total alkaloid (cevadine + veratridine) levels was performed on two seed lot populations, St-4.1, St-10.1, along with comparison *S. texanum* (St-28.1) and *S. officinale* (Venezuela) population samples (n = 1 seed lot or rep.). Sabadilla seeds were tested at a commercial laboratory under proprietary, in-house protocols developed by McLaughlin Gormley King Company’s (MGK) analytical chemistry team [4]. Seed samples were cryomilled, extracted in methanol, and processed using sonication and high-shear mixing; extracts were filtered immediately prior to vialing for analysis. Seed samples after cryomilling were stored at −15 °C until testing to avoid degradation. Quantification was performed using high-pressure liquid chromatography (HPLC) equipped with a ZORBAX Eclipse, XDB-C18, 5 µm, C-18 reverse phase column (Part Number: 993967-902; https://www.agilent.com/store/en_US/Prod-993967-902/993967-902 accessed 27 July 2026), 150 × 4.6 mm, and a UV detector. The HPLC-grade reagents included methanol (MetOH), acetonitrile, potassium hydrogen phosphate (K_2_HPO_4_), potassium dihydrogen phosphate (KH_2_PO_4_), and phenyl benzoate. The HPLC conditions in the gradient mobile phase were a MetOH buffer (25 mM solution of potassium phosphate filtered with a 0.45–0.5 µm filter, buffered to pH = 6.5 with KOH/H_3_PO_4_). The flow rate was 1.0 mL/min with the column heated (35 °C); wavelengths were VWD 240 nm (veratridine, cevadine, phenyl benzoate) and DAD 264 nm (veratridine). Isolated and purified veratridine and cevadine served as source standards. Calibration was based on a single-point external standard and an internal standard for seed assays to accommodate volume-related variability. Ten µl of standard solution was injected into the HPLC instrument until acceptable reproducibility was obtained. Tested samples were injected in a similar manner with only the peak area validated. Analytical quality control consisted of duplicated injections for every sample (n = 2 reps./sample).

**Figure 6:**
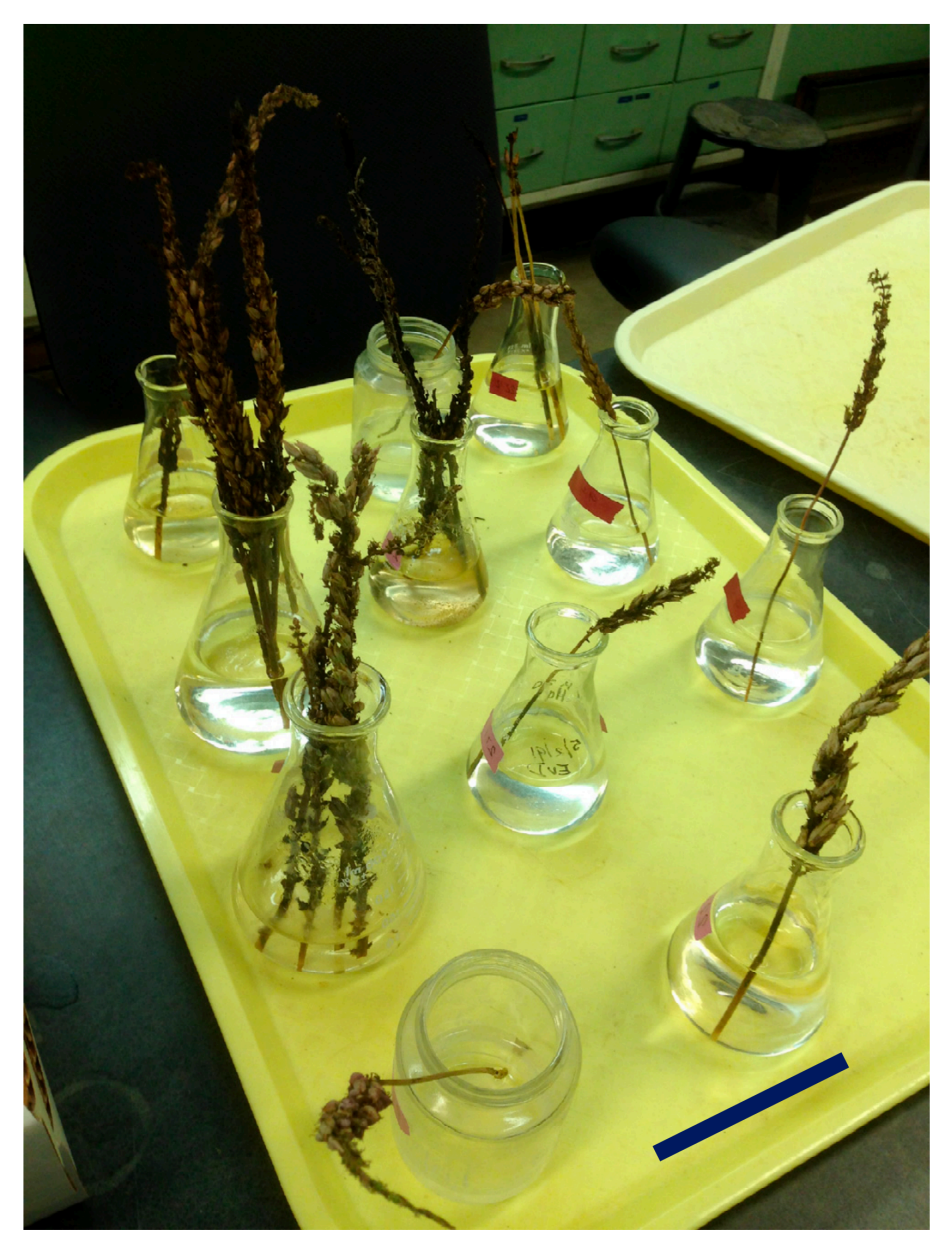
Laboratory post-harvest seed ripening of *Schoenocaulon texanum* racemes. Photo credit: Neil Anderson. Scale bar = 9 cm.

Acropetal flowering, pollination, and sequential seed ripening in *S. texanum* prevented harvest of mature capsules and seed at one time. Since we had funding for only one collection trip for two people over a 7-day period, we lacked sufficient funding to conduct multiple weekly collection trips during the ~3-month seed ripening period, preventing collection of each seed capsule as they individually matured. Thus, we needed to ripen the seed capsules ex situ to enable seed germination testing of mature seeds from all plants. Inflorescences (racemes) were harvested from each plant at the time of collection, tagged with plant identifiers (Table 9), bagged in resealable bags, and placed in a cooler for transport back to the laboratory. Upon arrival in the laboratory, racemes from each plant were placed into separate 250 mL Erlenmeyer flasks and filled with floral preservative solution [62] to supply carbon (sucrose) along with a bactericide to terminate the ripening and maturation of all capsules (Figure 6). This method produces viable seeds and has been used in many other crops [62], effectively removing source/sink interactions and maximizing seed ripening and maturation. Day/night temperatures in the laboratory were 21 ± 1 °C. Inflorescences remained in place in the laboratory for 2–4 weeks until all capsules were ripened and seed was mature for harvesting. Thereupon, seed data collected included the number of seed capsules/plant, mean number of capsules/inflorescence, total number of seeds produced/plant, number of seeds/inflorescence, number of seeds/capsule, seed dimensions (length/width, mm) and 100 seed weight (g).

Seeds of the tested germplasm were “cleaned” to the extent of stem or inflorescence stem tissue removal, enabling the seeds to serve as individual units for seed germination assessments. Seeds were then air-dried for 2 weeks at 21 °C (constant). No further cleaning or seed treatments were administered prior to conducting the seed germination tests. Seeds were packaged into seed lots on a per-plant basis within each population and stored dry in sealed containers in a walk-in seed vault for orthodox seed (3–5 °C, constant) until commencement of the seed germination experiments and alkaloid testing (52 weeks duration). Seed moisture content could not be determined, either pre- or post-storage, due to the low seed quantity available. Since the standard orthodox seed vault maintains low seed moisture levels (<10%), the assumption was that seed moisture contents most likely were in that range. A seed lot was defined as an open-pollinated population of half-sib individual seed units with genetically distinct capacity to germinate and results in a mature genotype [57,63]. The required amounts of seed were taken from the seed vault inventory on the day each seed germination experiment commenced. Bulk seed samples of two populations (Table 9) were quantified for alkaloid levels of cevadine (%) and veratridine (%) on a weight basis using standard extraction and chemical analyses [8,9].

### 4.3. Standard Seed Germination Experiment

As much as possible, seed germination tests were conducted via standard protocols of the Association of Official Seed Analysts, AOSA [39] at the Minnesota Crop Improvement Association (MCIA) laboratories, St. Paul Campus, University of Minnesota, St. Paul, MN USA. The Registered Seed Technologist (Mr. Chase Mowry, RST Seal number 161) coordinated the seed germination testing with sabadilla researchers for experimental setup, use of chambers, germination evaluations, and all other necessary equipment and facilities at MCIA.

Free-flowing seed samples were taken from each OP seed lot, although the complexities of sampling of large, commercial seed lots could not be followed due to the inherently small seed lot size. Instead, the sampling protocols for “seed in small containers (such as packets, tapes, mats, and small package lawn seed)” were used. As much as possible, the required minimum sample weights for “Vegetable and flower seed samples (as categorized by the AOSA Rules for Testing Seeds Vol. 3. Uniform Classification of Weed and Crop Seeds)” were followed [39], constituting the working sample, although the minimum of “at least 400” seeds was only possible in seed lot StG-1 whereas only one-half this amount (n = 198–200) was testable for seed lots St-1–St-9. Each seed lot replication had n = ~100 seeds, if available. These issues were unavoidable due to the quantity of seed samples available for harvest in the wild at the time of collection (Table 9). Upon arrival at the MCIA seed laboratory, the seeds were re-examined, although all seed was hand-carried <1 km across campus from the Anderson research laboratory to MCIA facilities.

Seed purity analytics were not conducted since the seed lots were wild-harvested rather than commercially harvested. Since all samples were direct-harvested from wild plants, this eliminated the need for determining the standard purity composition and/or the rate of occurrence of noxious weed seeds per unit weight. Thus, the percentage seed germination of pure raw seed (whole seeds with no selection bias; randomly counted), being free of any applied materials, was tested. Since only a seed germination test was required (no purity testing) and the pure seed fraction was >98%, pure seed working samples were taken using the “hand mixing/spoon method” as much as possible [39]. In summary, the official Laboratory Report of Analyses for each seed lot tested states: “Sampled not in accordance with AOSA rules. Analyzed according to Local procedures” [39]. Since neither *S. texanum* nor any related taxa within the genus *Schoenocaulon* have standards established for working seed sample weights, seed number was used in place thereof. The approximate number of seeds per gram was predetermined to guide future working sample weights. Standard, replicated germination tests (n = 2 replications) were conducted at 20 °C for 35 days during weeks 46 (2018)–4 (2019) in an incubated refrigerator (Model: NS146SISS, Norlake Scientific, Hudson, WI USA; https://norlakescience.com/ accessed on 27 July 2026). A standard comparison germination test was conducted with 400 seeds tested (StG-1, 18-00516 seed lot; n = 4 replications, 100 seeds/rep.) for comparison with each seed lot. Only seed lot StG-1 contained enough seed to perform a n = 400 seed test, as outlined for standard germ tests in the AOSA Rules [39].

**Figure 7:**
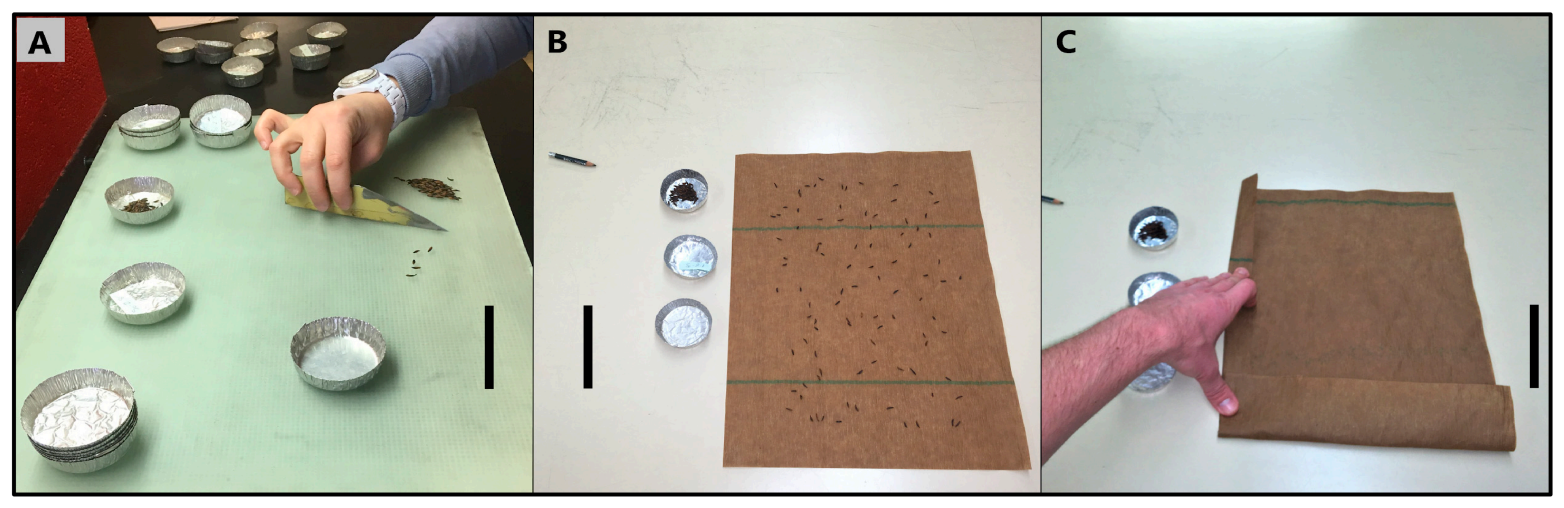
(**A**) Seed counting *Schoenocaulon texanum* seed lot replications, (**B**) sowing the seeds on a wet germination paper, and (**C**) covering the seeds with another germination paper, wrapping and folding up the papers. Photo credit: Michele Schermann. Scale bar: 10 cm.

**Figure 8:**
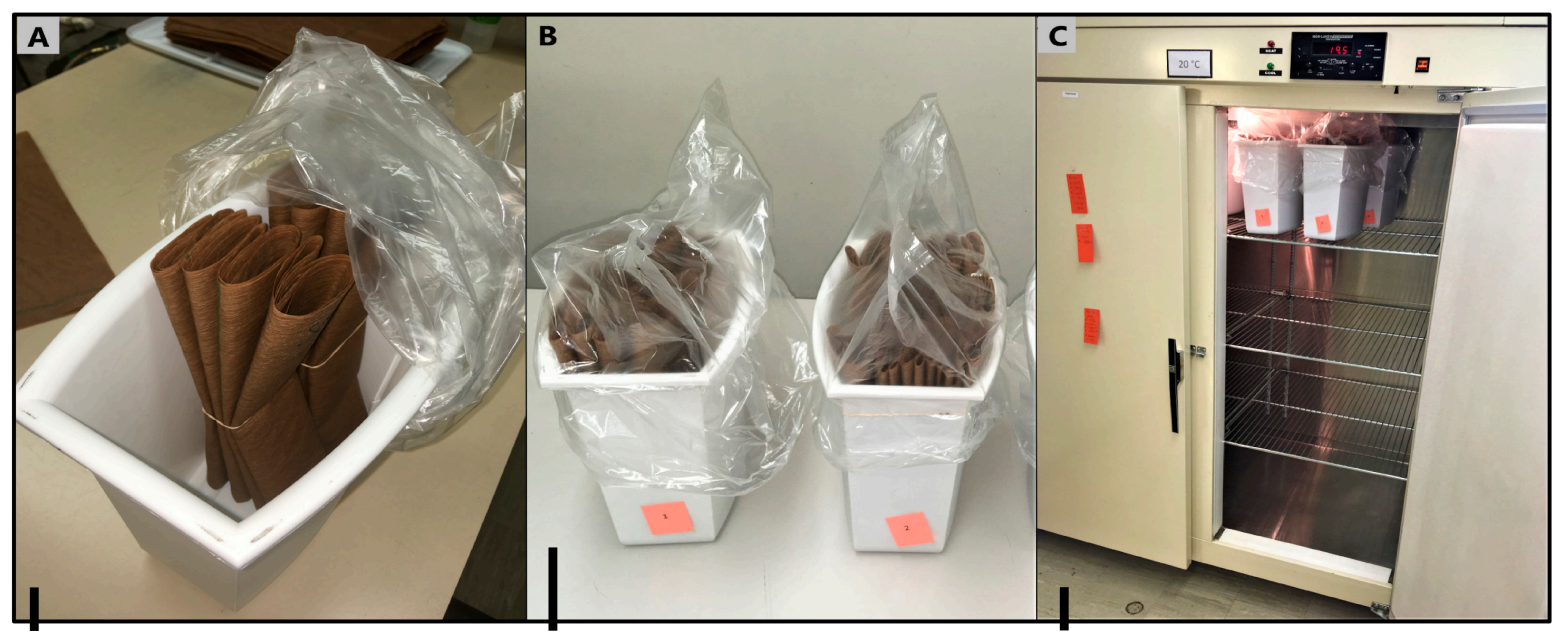
(**A**) Wrapped germination papers for standard seed germination of *Schoenocaulon texanum* seed lot replications, (**B**) germination papers in plastic containers covered with plastic bags to prevent them from drying out, and (**C**) seed lots in the germination chamber. Photo credit: Michele Schermann. Scale bar = 10 cm.

Each working sample (replication) was counted (Figure 7A), spread across a moistened standard germination testing paper, 48.5 × 36.5 cm heavy weight seed germination paper (SD7606, S 311M 76#; Anchor Paper Co., Saint Paul, MN, USA; https://www.anchorpaper.com/paper accessed on 27 July 2026) (Figure 7B), covered with another moistened germination paper (Figure 7C), and subsequently folded (Figure 7C). Sample replicates were secured together with a rubber band and then placed vertically in containers with a base solution of water (Figure 8A); the containers were covered with plastic (Figure 8B) and placed into the germination chamber (Figure 8C).

**Figure 9:**
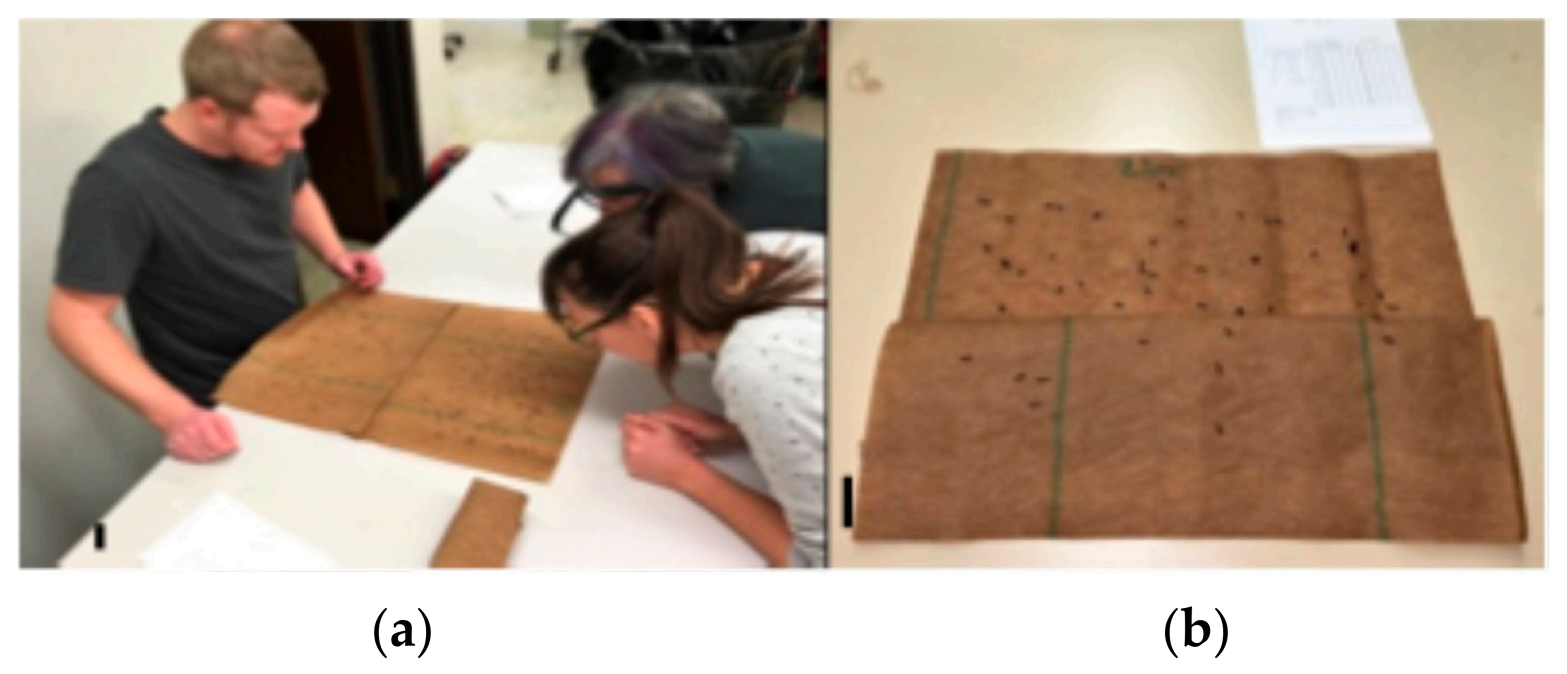
(**a**) *Schoenocaulon texanum* germination seedling counting at the Minnesota Crop Improvement Association laboratories, (**b**) an open germination paper with various stages of seed germination. Photo credits: Neil Anderson, Michele Schermann. Scale bar = 10 cm.

Seed germination was defined as “emergence and development from the seed embryo of those essential structures that, for the kind of seed in question, are indicative of the ability to produce a normal plant under favorable conditions” [39]. Germination papers were checked weekly during the six germination weeks (G1–G6) and the seed germination was evaluated/counted according to AOSA definitions for normal seedlings, abnormal seedlings, hard seeds, dead seeds, or potentially dormant seeds (Figure 9a,b). Since TZ tests were not conducted, dormancy status of non-germinated seeds could not be determined or quantified.

### 4.4. Saturated Salt Accelerated Aging (SSAA) Seed Germination Experiment

Since neither long-term storage seed viability nor accelerated aging vigor tests have been conducted in any *Schoenocaulon* taxa, a best practices method was adopted from other small-seeded crops, e.g., onion, lettuce, and rice [39,42] for testing *S. texanum* in this experiment. Saturated salt accelerated aging tests were performed at the Minnesota Crop Improvement Association (MCIA), where aging chambers and germination cabinets were used that met the exact requirements of temperature and relative humidity, to make accurate and repeatable estimates: 76% RH (NaCl) SSAA test conducted at 45 °C for 72 h. [39,40,64,65]. Following the SSAA treatment, seeds were germinated under standard conditions at 20 °C for 35 days. Seed sampling for this experiment was the same as for standard seed germination (n = ~100 seeds/rep. within seed lots, if available). The SSAA test consisted of testing n = 198–200 seeds/lot for seed lots of St-1 through St-9. The tests (successive runs; 27 boxes/run) consisted of n = 99–100 seeds/box and two replications. Higher quantities could not be tested due to the lack of seed availability and low numbers of plants and open-pollinated seeds available for collection.

**Figure 10:**
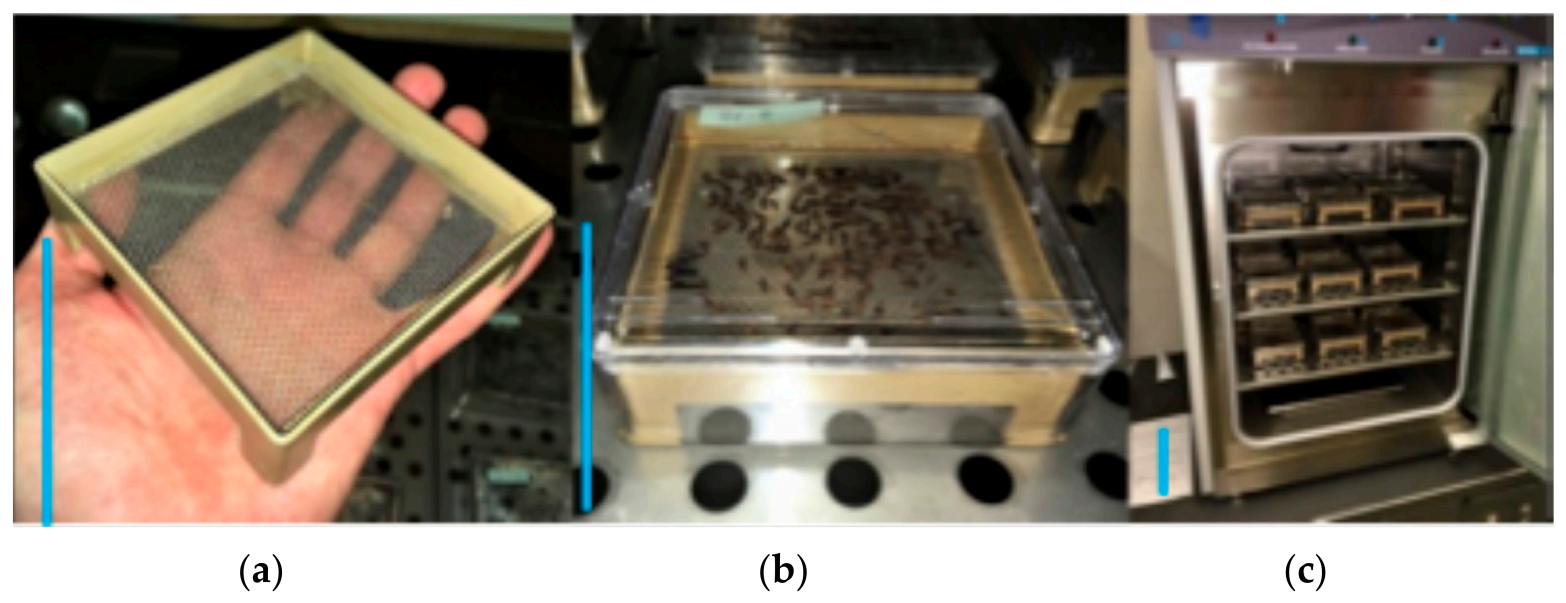
Elevated screen (**a**) for holding the *Schoenocaulon texanum* seeds during the saturated salt accelerated aging (SSAA) test, (**b**) acrylic box containing the seeds and the NaCl saline solution, and (**c**) the SSAA chamber. Photo credits: Michele Schermann. Scale bar = 11 cm.

Seeds were spaced out evenly on an elevated screen (Figure 10a), 40 mL of a saturated NaCl solution was poured into each acrylic box (11 × 11 × 3.5 cm, lxwxh), and then the screens were placed into the boxes and covered with lids (Figure 10b). The prepared boxes were placed in a water-jacketed CO_2_ incubator or SSAA chamber (Model: SCO5W, Sheldon Manufacturing Inc., Cornelius, OR USA; https://www.sheldonmanufacturing.com/shel-lab-products/productid/SCO5W accessed on 27 July 2026) maintained at 45 °C for 72 h (Figure 10c). The CO_2_ capability was not used in this study. The NaCl solution maintained ~76% RH inside the boxes.

The first SSAA run was finished in week 50 (on 14 December) and the second run terminated in week 51 (on 21 December). The seeds of both runs were transferred to pre-moistened 48.5 × 36.5 cm heavyweight seed germination paper (SD7606, S 311M 76#; Anchor Paper Co., Saint Paul, MN, USA; https://www.anchorpaper.com/paper accessed on 27 July 2026) and then placed into a germination chamber that maintained the temperature at 20 °C (Figure 8C). During each week, for six weeks (G1–G6), the germination papers were checked, and seedlings were evaluated/counted according to AOSA criteria used for standard seed germination tests.

### 4.5. Data Analyses

Percent seed germination data were calculated [66] and arcsin transformed prior to conducting Analysis of Variance (ANOVA) and mean separations using Tukey’s Honestly Significant Difference (HSD) test at α = 0.05 [66]. Correlations of all traits were also performed. The Statistical Package for the Social Sciences (SPSS) v. 29.0.1.0 (S. Chicago, IL, USA) was used for all analyses.

## 5. Conclusions

Soil constituents of the native soils were extrapolated for potential commercial production conditions (high pH levels, low EC, moderate N levels, high porosity) of *S. texanum*. Accumulation of toxic heavy metals in a potential green pesticide product could be alleviated with molecular techniques such as SNP markers, gene editing or phytomineralization to sustainably detoxify soils. Alkaloid (cevadine, veratridine) levels were ~20% lower than in a related commercial relative, *S. officinale*, due to a ~30% reduction in seed size. While *S. texanum* seeds are smaller than *S. officinale*, both share appearance, hardness, color and shape similarities. Wider sampling of *S. texanum* populations and/or focused breeding may improve this. Twelve is the maximum number of bulbs/clump in a mature bulb cluster, each with the potential to generate 1–2 inflorescences/mature bulb. Standard and SSAA germination begins three weeks (G3) after sowing and continues through week G6 in this experiment. The highest % germination occurred in weeks G3–G4. We establish initial seed germination standards of ≥70% for both standard and SSAA germination tests, based on total or adjusted final % germination of seed lots. Limitations of this study relate to the limited number of populations and genotypes that could be collected in the wild and low seed set which reduced the number of samples for all quantitative tests. This is common for studies involving wild plant species without any previous research, using genotypes sampled from extant populations. Future research would provide data for potential commercialization of this species as a green pesticide crop.

## Figures and Tables

**Table 1:** Soil test results from the Spurway system, Exchangeable ion method and Microwave digest with HNO_3_ ICP (pH, organic matter, electrical conductivity or EC, macro- and micro-nutrients) analyses for field samples collected in extant *Schoenocaulon texanum* populations (seed lots: St-1, St-2, St-4, St-7, St-8, St-9) during the plant collection trip in Texas (2016). Means and ranges (min.–max.) are pooled for all soil collection population sites used to generate the six seed lots. Greenhouse standard for the Spurway system tests are included for comparative purposes.

Parameters	Pooled Means (Range)			Spurway System Greenhouse Standard ^2^
	Spurway System	Exchangeable Ion Method	Microwave Digest with HNO_3_ ICP Analyses	
Organic Matter	4.6 (1.9–7.1)%			33–100%
pH	8.05 (7.8–8.2)			6.2–6.5
EC, 1:1	0.2 (0.2) mmhos/cm			1.2–1.8
**Macronutrients**				
NH_4_N ^1^	1.39 (0.96–2.9) ppm			n/a
NO_3_ N ^1^	2.33 (0.72–4.57) ppm			150–180 ppm
Bray P	n/a ^3^			n/a ^3^
Olsen P	2.5 (1–4) ppm			5–10 ppm
NH_4_OAc-K ^1^	85 (79–356) ppm	157.84 (71.63–302.68) ppm, exchangeable		50–60 ppm
NH_4_OAc-Ca		6151.8 (4442.4–8028.9) ppm		120–180 ppm
NH_4_OAc-Mg		100.9 (29.1–182.8) ppm		40–60 ppm
SO_4_−S	3.5 (3–4) ppm			n/a
**Micro-nutrients**				
DTPA-Fe		5 (3.1–6.5) ppm		0.25–0.5 ppm
DTPA-Mn		6.4 (2.9–10.7) ppm		0.25–0.5 ppm
DTPA-Zn		0.98 (1.39–2.69) ppm		0.25–0.5 ppm
DTPA-Cu		0.41 (0.24–0.65) ppm		n/a
NH_4_OAc-Na, exchangeable		4.54 (3.1–8.1) ppm		n/a
B		0.49 (0.35–0.78) ppm	23.6 (13.29–42.46) ppm	0.25–0.5 ppm
**Heavy Metals**				
Al			19,958 (6126–35,820) ppm	0 ppm
Cd			1.8 (0.64–3.32) ppm	0 ppm
Cr			16.5 (6.5–28.2) ppm	0 ppm
Ni			11.4 (4.1–20.1) ppm	0 ppm
Pb			12.4 (6.9–18.2) ppm	0 ppm

^1^ Nitrate N and Ammonium N results have been corrected for sample moisture content, reported on dry-weight equivalent basis. ^2^ https://soiltest.cfans.umn.edu/interpretation-soil-test-results-horticultural-crops (accessed on 1 March 2026). ^3^ Only Olsen P is applicable, since the pH > 7.4 (https://soiltesting.cahnr.uconn.edu/interpretation-of-sme-results-for-greenhouse-media/ (accessed on 1 March 2026)).

**Table 2:** Physical properties (seed weight, mg; H_2_O %*w*/*w*; oil (lipids) %*w*/*w*) and alkaloid content (cevadine, veratridine, ratio of cevadine: veratridine, combined alkaloids) of three tested *Schoenocaulon texanum* populations with a comparison species, *S. officinale*, the commercial standard (n = 1 seed lot tested for each sample, due to limited seed).

Taxa, Seed Lot No. (Population.genotype) Codes	Seed Weight (mg)	H_2_O %*w*/*w*	Oil (Lipids) %*w*/*w*	Cevadine Content (%)	Veratridine Content (%)	Ratio of Cevadine:Veratridine (C:V)	Combined Alkaloids (%)
*S. texanum*, St-1 (28.4)	5.2	6.1	15.9	0.51	0.29	1.8	0.80
*S. texanum*, St-2 (4.1)	3.3	6.2	17.2	0.43	0.27	1.6	0.70
*S. texanum*, St-6 (10.1)	5.0	6.1	17.0	0.03	0.29	0.1	0.31
*S. officinale*, commercial standard ^1^	6.3	8.0	20.0	2.00	1.20	1.6	3.60

^1^ Seed lot supplied by McLaughlin Gormley King Company which was collected in Venezuela (passport information undisclosed.

**Table 3:** Seed production parameters for the open-pollinated seed lots collected from ten extant genotypes of *Schoenocaulon texanum* in the State of Texas. Bulb and inflorescence data (number of bulbs/clump, number of flower stems/plant) were collected in situ on the plants while the seed parameters were collected after laboratory seed ripening and included the number of seed capsules/plant, number of seeds produced, number of capsules/inflorescence, number of seeds/inflorescence, number of seeds/capsule, mean 100 seed weight (g), mean seed length (mm) and mean seed width (mm). Mean separations within columns denoted with letters, using Tukey’s Honestly Significant Difference (HSD) test at α = 0.05.

Seed Lot No. (Population.genotype Code)	Mean No. Bulbs/Clump	Mean No. Flw. Stems/Plant	Total No. Seed Capsules/Plant	Total No. of Seeds Produced/Plant	Mean No. of Capsules/Inflorescence	Mean No. Seeds/Inflorescence	Mean No. Seeds/Capsule	Mean 100 Seed Weight (g)	Mean Seed Length (mm)	Mean Seed Width (mm)	Mean Seed Length:Width (l:w) Ratio
St-1 (28.4)	4	5	209 b	410	41.80 b	82.00	1.96	0.55	4.81 bc	0.49 a	9.82 c
St-2 (4.1)	11	4	81 a	294	20.25 a	73.50	3.63	0.28	4.63 abc	0.9 b	5.14 b
St-3 (4.3)	2	2	52 a	356	26.00 a	178.00	6.85	0.31	4.3 abc	0.9 b	4.78 b
St-4 (4.9)	12	8	126 a	593	42.00 b	197.67	4.71	0.46	3.9 abc	1.27 b	3.07 a
St-5 (4.10)	5	1	59 a	203	59.00 b	203.00	3.44	0.25	3.95	1.25 b	3.16 ab
St-6 (10.1)	4	3	46 a	349	15.33 a	116.33	7.59	0.55	3.7 ab	1.25 b	2.96 a
St-7 (10.5)	2	2	32 a	238	16.00 a	119.00	7.44	0.28	4.25 ab	1.25 b	3.40 ab
St-8 (22.2)	3	3	104 a	375	33.00 ab	125.00	3.61	0.23	4.95 c	1.0 b	4.95 b
St-9 (22.7)	3	7	92 a	258	13.14 a	36.86	2.80	0.44	5.4 c	1.15 b	4.69 b
StG-1 (20.1)	7	5	185 b	657	37.00 ab	131.40	3.55	0.33	3.55 a	1.3 b	2.73 a
Pooled means	4.34	4	---	373.3	---	---	4.56	0.45	---	---	---

**Table 4:** Pearson’s correlation (r) matrix for morphological traits of ten *Schoenocaulon texanum* seed lots.

	No. of Bulbs/Clump	Mean No. Flw. Stems/Plant	Total No. Seed Capsules/Plant	Total No. of Seeds Produced/Plant	Mean No. of Capsules/Inflorescence	Mean No. Seeds/Inflorescence	Mean No. Seeds/Capsule	Mean 100 Seed Weight (g)	Mean Seed Length (mm)	Mean Seed Width (mm)
No. of bulbs/clump	1.0									
Mean no. flw. stems/plant	0.505	1.0								
Total no. seed capsules/plant	0.224	0.556	1.0							
Total no. of seeds produced/plant	0.477	0.545	0.682	1.0						
Mean no. of capsules/inflorescence	−0.265	0.072	−0.169	−0.026	1.0					
Mean no. seeds/inflorescence	−0.258	−0.043	−0.394	0.053	−0.169	1.0				
Mean no. seeds/capsule	−0.226	−0.400	−0.422	0.005	0.174	0.699 ^1^	1.0			
Mean 100 seed weight (g)	−0.013	0.515	0.422	0.166	−0.184	−0.130	−0.426	1.0		
Mean seed length (mm)	−0.347	0.165	0.018	−0.528	0.018	−0.528	−0.519	0.123	1.0	
Mean seed width (mm)	0.214	−0.041	−0.445	0.170	0.107	0.642 ^1^	0.773 ^2^	−0.424	−0.607 ^1^	1.0

^1^ *p* ≤ 0.05. ^2^ *p* ≤ 0.01.

**Table 5:** Standard germination tests for five extant, wild populations, consisting of ten seed lots, of *Schoenocaulon texanum* (cf. Table 1) conducted at the Minnesota Crop Improvement Association (MCIA): seed lot and MCIA lab test number codes, number (N) of seeds tested, number of seeds/g, weight in (g), weight out (g), weight difference (weight out—weight in; g), number seeds germinated, percent (%) germination for germination (G) weeks G3, G4, G5, and G6; final % germination, adjusted final % germination. Each seed lot was divided equally into two replications prior to testing. Note: there was zero percent (0%) germination in weeks G1–G2 for all seed lots tested. Percent seed germination data were arcsin transformed prior to conducting Analysis of Variance; mean separations within columns are denoted with letters, using Tukey’s Honestly Significant Difference (HSD) test at α = 0.05.

Seed Lot, MCIA Lab Test No.	No. Sds.	No. Sds./g	Wt. In (g)	Wt. Out (g)	Wt. Diff. (g)	G3		G4		G5		G6		Final % Germ	Adjusted Final % Germ
						No. Germ	% Germ	No. Germ	% Germ	No. Germ	% Germ	No. Germ	% Germ		
St-1, 18-00507	200	182	1.1 e	1.2 f		74	37 bc	50	25 b	17		7	4 ab	74.0	74 fg
St-2, 18-00508	200	357	0.56 ab	0.59 abc		61	31 abc	20	10 ab	4		4	2 ab	44.5	45 d
St-3, 18-00509	199	321	0.62 b	0.65 c		88	44 bc	15	8 ab	2		1	1 a	62.3	62 e
St-4, 18-00510	200	217	0.92 d	1.0 e		153	77 d	21	11 ab	5		2	1 a	90.5	91 h
St-5, 18-00511	200	400	0.5 a	0.56 ab		64	32 abc	8	4 a	3		3	2 ab	39.0	39 c
St-6, 18-00512	200	183	1.09 e	1.18 f		111	56 cd	36	18 ab	3		5	3 ab	77.5	78 g
St-7, 18-00513	199	355	0.56 ab	0.62 bc		13	7 a	7	4 a	4		3	2 ab	13.6	14 a
St-8, 18-00514	198	440	0.45 a	0.49 a		38	19 ab	23	12 ab	5		4	2 ab	32.8	33 b
St-9, 18-00515	200	225	0.89 d	0.96 e		67	34 abc	17	9 ab	2		3	2 ab	44.5	45 d
StG-1, 18-00516	400	300	0.75 c	0.80 d		182	46 bc	79	20 ab	28		20	5 b	72.0	72 f
Pooled		268			0.06						3.2			55.1	

**Table 6:** Correlation matrix (Pearson’s *r* values) for standard germination tests of ten seed lots derived from five extant, wild populations of *Schoenocaulon texanum* (cf. Table 1) conducted at the Minnesota Crop Improvement Association (MCIA) for variables: weight in (g), weight out (g), weight difference (weight out—weight in; g), number seeds germinated, percent (%) germination for germination (G) weeks G3, G4, G5, and G6 and adjusted final % germination.

	Weight In (g)	Weight Out (g)	Weight Diff. (g)	G3% Germ.	G4% Germ.	G5% Germ.	G6% Germ.	Adjusted Final % Germ
Weight in (g)	1.0							
Weight out (g)	0.99 **	1.0						
Weight diff. (g)	0.35	0.45 *	1.0					
G3% germ.	0.54 *	0.59 **	0.44	1.0				
G4% germ.	0.62 *	0.57 **	−0.10	0.18	1.0			
G5% germ.	0.26	0.26	0.19	0.06	0.43 *	1.0		
G6% germ.	0.28	0.22	−0.34	−0.01	0.69 **	0.43 *	1.0	
Adjusted final germ	0.74 **	0.74 **	0.28	0.89 **	0.59 **	0.28	0.29	1.0

* *p* ≤ 0.05; ** *p* ≤ 0.01.

**Table 7:** Saturated salt accelerated aging (SSAA) germination tests for five wild populations of *Schoenocaulon texanum* (cf. Table 1) at the Minnesota Crop Improvement Association (MCIA): seed lot and MCIA lab number codes, number (N) of seeds tested, number seeds germinated, percent (%) germination for germination (G) weeks G3, G4, G5, and G6; final % germination, adjusted final % germination. Each seed lot was divided equally into two replications prior to testing. Note: zero percent (0%) germination in weeks G1–G2 for all seed lots tested. Percent seed germination data were arcsin transformed prior to conducting Analysis of Variance; mean separations within columns denoted with letters, using Tukey’s Honestly Significant Difference (HSD) test at α = 0.05.

Seed Lot, Lab No.	No. Sds.	G3		G4		G5		G6		Final % Germ	Adjusted Final % Germ
		No. Germ	% Germ	No. Germ	% Germ	No. Germ	% Germ	No. Germ	% Germ		
St-1, 18-00507	200	74	37.0 d	50	25.0 e	17	8.5 e	7	3.5 f	74.0	71 f
St-2, 18-00508	200	61	30.5 c	20	10.0 c	4	2.0 bc	4	2.0 d	44.5	43 cd
St-3, 18-00509	199	88	44.2 e	15	7.5 b	2	1.0 a	1	0.5 a	53.2	53 e
St-4, 18-00510	200	153	76.5 g	21	10.5 c	5	2.5 d	2	1.0 b	90.5	90 h
St-5, 18-00511	200	64	32.0 c	8	4.0 a	3	1.5 ab	3	1.5 c	39.0	38 bc
St-6, 18-00512	200	111	55.5 f	36	18.0 d	3	1.5 ab	5	2.5 e	77.5	75 f
St-7, 18-00513	199	13	6.5 a	7	3.5 a	4	2.0 bcd	3	1.5 c	13.6	12 a
St-8, 18-00514	198	38	19.2 b	23	11.6 c	5	2.5 cd	4	2.0 d	35.4	33 b
St-9, 18-00515	200	67	33.5 c	17	8.5 b	2	1.0 a	3	1.5 c	44.5	48 de

**Table 8:** Correlation matrix (Pearson’s *r* values) for saturated salt accelerated aging (SSAA) germination tests of ten seed lots derived from five extant, wild populations of *Schoenocaulon texanum* (cf. Table 1) conducted at the Minnesota Crop Improvement Association (MCIA) for variables: percent (%) germination for germination (G) weeks G3, G4, G5, G6 and adjusted final % germination.

	G3% Germ.	G4% Germ.	G5% Germ.	G6% Germ.	Adjusted Final % Germ
G3% germ.	1.0				
G4% germ.	0.30	1.0			
G5% germ.	0.01	0.78 **	1.0		
G6% germ.	−0.15	0.82 **	0.74 **	1.0	
Adjusted final % germ	0.93 **	0.61 **	0.31	0.17	1.0

** *p* ≤ 0.01.

**Table 9:** Extant population collection information for five wild, extant populations of *Schoenocaulon texanum* growing in the State of Texas, U.S.A. used for seed germination experiments: herbarium, sample identifiers; seed lot codes; population codes (number of genotypes); county, State of Texas; latitude N (decimal), longitude W (decimal), mean number of bulbs/plant; mean number of flower stems/plant; total number of seed capsules/plant; total number of seeds collected; soil sample collected (S), alkaloid tested (A). Genotypes (n = 10) were selected in this study, based on seed quantities available (N = 198–200, cf. Table 2), and evaluated to set *S. texanum* seed germination standards for standard and accelerated aging tests.

Herbarium, Sample Identifier	Seed Lot Codes	Pop’n. Code (No. Genotypes)	State of Texas County	Lat. N	Long. W	Soil Sample Collected (S), Alkaloid Tested (A)
---	St-1	28.4 (1)	Bandera	29.6914	−99.1725	S, A
University of Texas, Austin Herbarium, TEX00196805, 1291489	St-2	4.1 (4)	Hays	30.1406	−98.0317	S, A
	St-3	4.3 (1)				
	St-4	4.9 (1)				S
	St-5	4.10 (1)				
New York Botanical Garden, Catalog #88161	St-6	10.1 (1)	Edwards	30.2569	−99.9503	A
	St-7	10.5 (1)				S
University of Texas, Austin Herbarium, TEX00433237, 1307255	St-8	22.2 (1)	Kerr	30.1	−99.4939	S
	St-9	22.7 (1)				S
University of Arizona Herbarium, Catalog #405544	StG-1	20 (1)	Kimble	30.4695	−99.7751	

## Data Availability

The data that support the findings will not be available due to an embargo from the date of publication to allow for commercialization of research findings.

## Abbreviations

The following abbreviations are used in this manuscript:

AA	Accelerated Aging
G	Germination week
HPLC	High-Pressure Liquid Chromatography
MCIA	Minnesota Crop Improvement Association
RH	Relative Humidity
SSAA	Saturated salt accelerated aging
TTC	Triphenyl tetrazolium chloride
Z	USDA Winter Hardiness Zone
